# The role of collagen and collagen I/III ratio in pathological conditions: insights into molecular mechanisms and therapeutic approaches

**DOI:** 10.3389/fbioe.2025.1679625

**Published:** 2025-10-09

**Authors:** Hemalatha Kanniyappan, Rathnayake A. Chathurika Rathnayake, Jude Osamor, Maria Islam, Rong R. Wang

**Affiliations:** ^1^ Department of Chemistry and Biochemistry, Worcester Polytechnic Institute, Worcester, MA, United States; ^2^ Department of Chemistry, Illinois Institute of Technology, Chicago, IL, United States

**Keywords:** collagen, collagen I/III ratio, pelvic organ prolapse, ovarian cancer, periodontal disease, osteoporosis, wound healing

## Abstract

Collagen is the predominant structural protein, contributing to 25%–30% of total body protein. It is vital for maintaining the strength, flexibility, and structural integrity of connective tissues throughout the human body. Of the 28 identified collagen types, collagen I and collagen III are especially critical - collagen I imparts tensile strength, while collagen III enhances matrix flexibility. Disruptions in collagen structure and composition are frequently associated with aberrant collagen I and collagen III ratio that compromises tissue functions and contributes to pathological conditions affecting bone (osteoporosis), oral tissues (periodontal disease), wound healing (diabetic complications), reproductive organs (ovarian cancer), and pelvic support structures (pelvic organ prolapse), among others. These alterations arise from aging, genetic polymorphisms, and disease factors that disrupt collagen synthesis, assembly, and degradation. This review highlights recent advances in understanding the role of collagen and collagen I/III ratio in pathophysiological processes and deliberates emerging therapeutic interventions designed to restore collagen equilibrium, encompassing biomaterials, stem cell therapies, gene editing techniques, and biophysical stimulation modalities. Future directions in tissue-engineered extracellular matrix development, precision medicine applications, and combined therapeutic strategies are discussed as transformative approaches for managing collagen-associated disorders and improving patient outcomes.

## 1 Introduction

About 30% of human protein mass comprises collagen, the main structural protein in the extracellular matrix (ECM) (Karamanos et al., 2021; Zhou et al., 2024). Collagen is the major component of a variety of connective tissues, including skin, bone, tendon, cartilage, and blood vessels, offering the tissues’ tensile strength, structural stability, and elasticity (Huebner et al., 2020; Xu et al., 2021; Li et al., 2024). The distinctive triple-helical structure of collagen molecules consists of three α-chains arranged in a (Gly-X-Y)n pattern, and its biomechanical qualities depend on this arrangement (Mienaltowski et al., 2021; A. Gerrein et al., 2025). The two most common types of collagen are collagen I, found in bones, tendons, and most connective tissues, and collagen III, found in greater quantities in skin and blood vessels (Henriksen and Karsdal, 2024). Imbalance in the ratio of collagen I and collagen III disrupts the tissue’s homeostasis, resulting in a variety of inflammatory, neoplastic, and degenerative diseases (Singh et al., 2023).

Beyond its structural functions, collagen is crucial because it acts as a bioactive matrix that interacts with growth factors, signaling molecules, and integrins (Rinaldi et al., 2021; Surdiacourt et al., 2025). Collagen homeostasis is regulated through the coordinated balance between anabolic processes (fibroblast-mediated collagen synthesis and secretion) and catabolic processes (matrix metalloproteinase (MMPs)-mediated proteolytic cleavage of collagen fibrils), warranting proper extracellular matrix turnover and tissue integrity (Pires et al., 2022; FitzGerald et al., 2024). Dysregulation of collagen homeostasis has profound pathophysiological consequences that manifest across multiple organ systems and health conditions (Panwar et al., 2018; Jürgensen et al., 2020). Impaired tissue mechanics (Silver et al., 2021), slowed wound healing (Yen et al., 2018; Wang et al., 2021), increased vulnerability to fractures (Li et al., 2018), and even intensified tumor invasiveness, can all result from abnormal collagen synthesis, degradation, or structural organization (Beam et al., 2015; Liang et al., 2020). Notably, advanced imaging methods, including second-harmonic generation microscopy, have enabled label-free visualization and quantitative assessment of collagen fibers’ density, orientation, and 3D organization within intact tissues, which aid in determining how a disease or disorder is progressing and how effective a treatment works (Kim et al., 2016; Esquibel et al., 2020).

**FIGURE 1:**
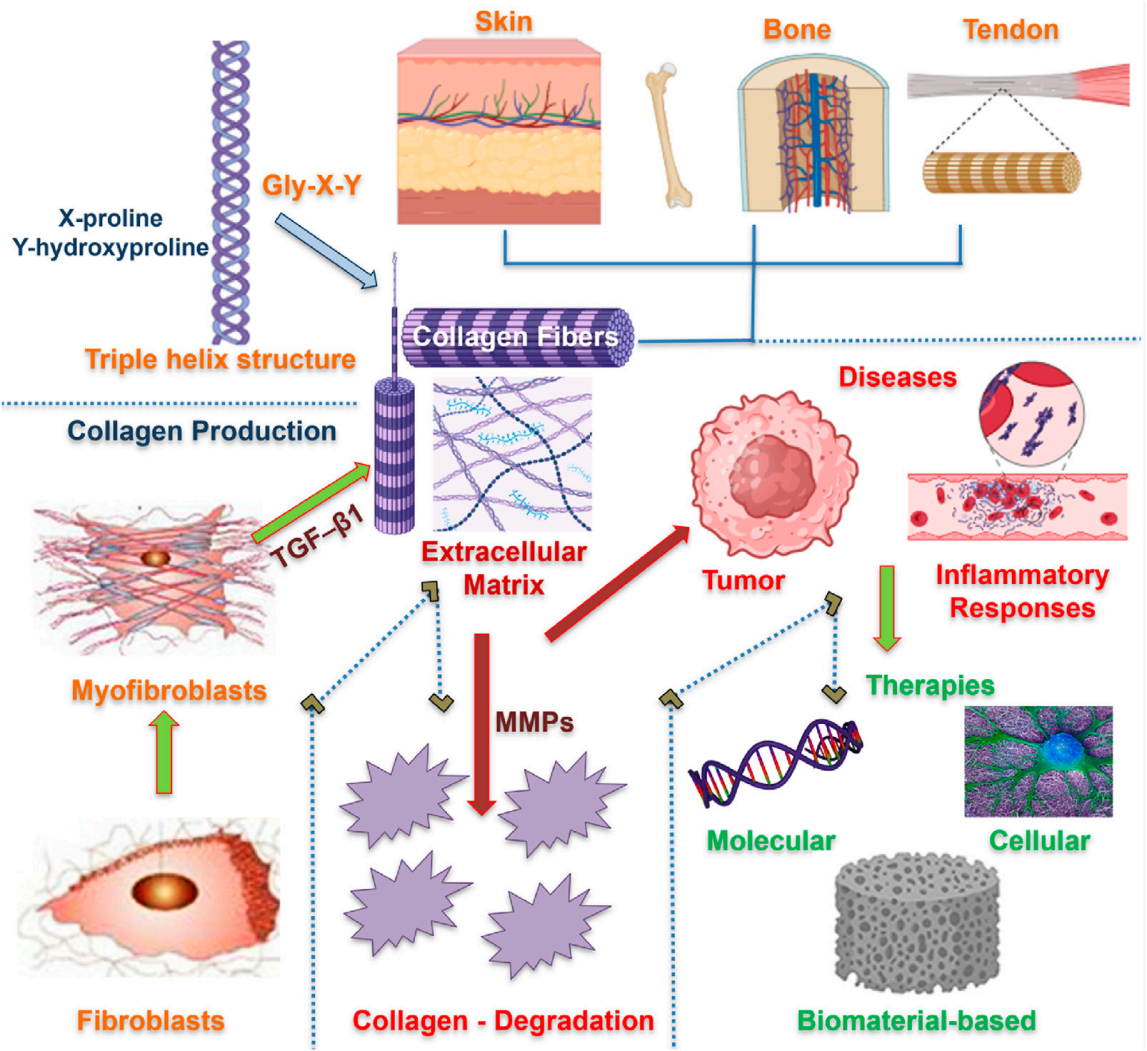
An outline of collagen biology, diseases, and therapies. Fibroblasts differentiate into myofibroblasts, leading to the synthesis of collagen and the formation of a triple-helical structure. The produced collagen fibrils are organized within the ECM matrix of different organs, providing structural integrity. Collagen imbalance and degradation are regulated by MMPs, responsible for several pathological conditions. Therapeutic approaches are devised to remodel collagen at molecular, cellular, and tissue levels.

This review provides a comprehensive understanding of collagen biology, identifies diseases caused by imbalances in the collagen I/III ratio, and presents new developments in therapeutic approaches that use molecular, cellular, and material-based techniques to restore tissue integrity, as depicted in Figure 1 **.**

## 2 Collagen biology and structure

### 2.1 Synthesis and structure

**FIGURE 2:**
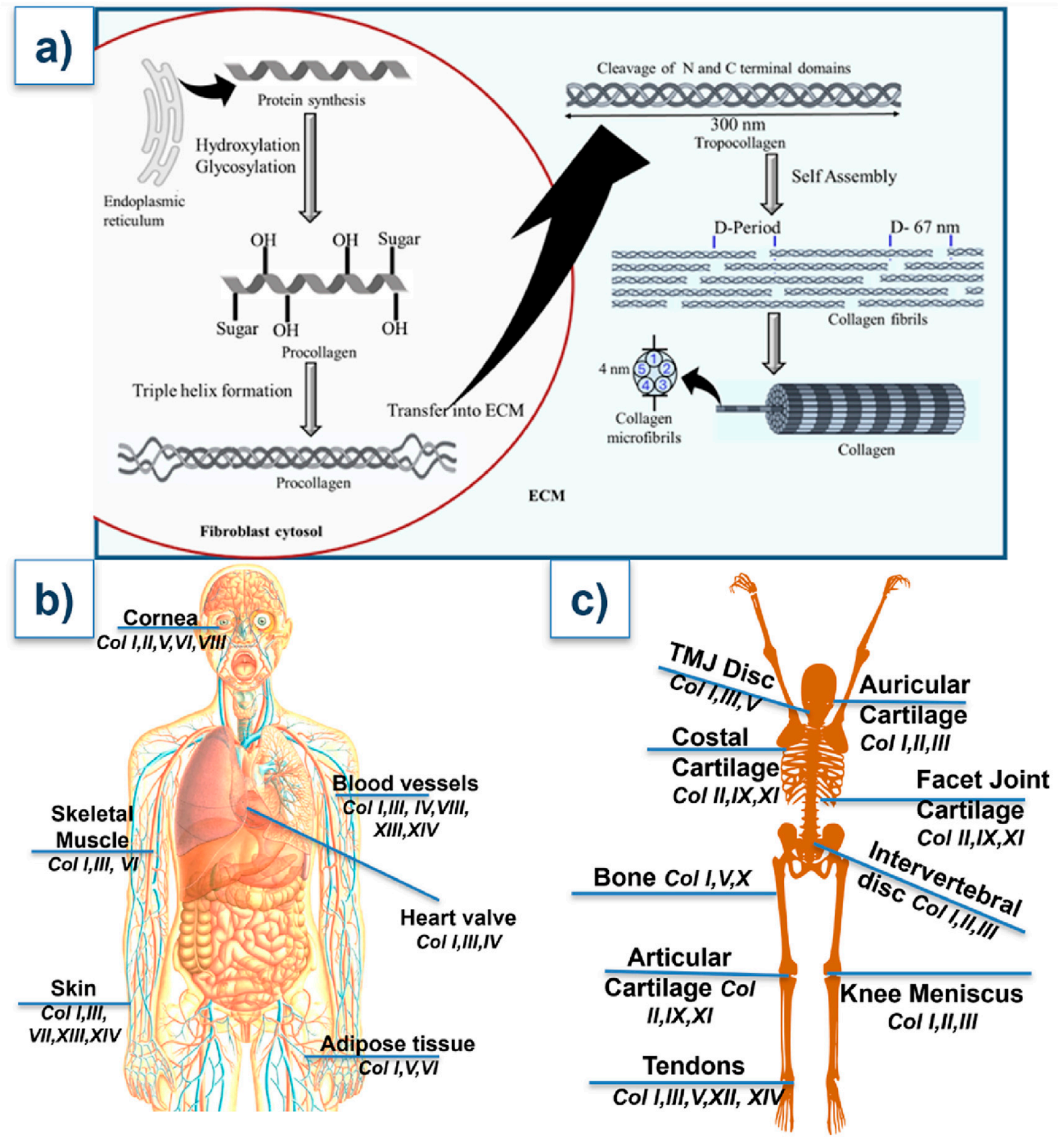
**(a)** Structural biology of collagen. **(b,c)** Types of collagens present at the different parts of the body. Their detailed functions are outlined in Table 1.

A highly synchronized process, collagen production starts in the endoplasmic reticulum of fibroblasts, osteoblasts, chondrocytes, and other cells that produce proteins of the extracellular matrix (Orgel et al., 2011; Narauskaitė et al., 2021). As shown in Figure 2a, translation of α-chain precursors is the first step in the formation of procollagen. Proline and lysine residues are then hydroxylated (a process that requires vitamin C), and hydroxylysine is glycosylated. These post-translational modifications are critical for collagen triple helix formation and stability (Yamauchi and Sricholpech, 2012).

After forming a triple helix, the procollagen molecule is released into the extracellular space, where its N- and C-terminal propeptides are broken down by specific enzymes (Perumal et al., 2008). The resultant tropocollagen molecules self-assemble into fibrils with a distinctive quarter-staggered pattern, called D-period (Giubertoni et al., 2024). Lysyl oxidase catalyzes cross-linking, which adds toughness and tensile strength to the fibrils. This multi-level assemblage governs collagen’s resistance to mechanical stress (Herchenhan et al., 2015). Collagen pathologies can result from diverse etiological factors that disrupt normal collagen synthesis, post-translational modification, or ECM assembly. These include genetic mutations, abnormal enzyme levels affecting collagen processing, nutritional deficiencies that impair cofactor availability for essential enzymatic reactions, or injuries (Lu et al., 2019).

### 2.2 Collagen types and functions

According to their structure and function, collagen comes in at least 28 distinct forms (Table 1). As illustrated in Figures 2b,c, large fibrils of collagen I, which is the most prevalent, are present in bone, skin, tendons, and ligaments. Collagen III typically forms hetero fibrils with collagen I. It offers distension and flexibility of tissues in the skin, blood vessels, and intestines. The collagen I/III ratio in the hetero-fibrils regulate the tissues’ elasticity and tensile strength (Kuivaniemi and Tromp, 2019; Wang J. et al., 2022). Other significant types of collagen include: collagen II- mainly presents in the eye’s vitreous body and cartilage, and is necessary for the integrity of cartilage (Lai et al., 2020); collagen IV- creates a network resembling a mesh in basement membranes, and is essential for the kidney glomerulus’ filtration processes and the epithelial tissues’ structural support (Bersie-Larson et al., 2020); collagen V and XI- participate in fibrillogenesis and control the fibril diameter (Wenstrup et al., 2011); collagen VII- causes dystrophic epidermolysis bullosa due to alterations in anchoring fibrils, which connect the dermis to the epidermis (Chung and Uitto, 2010).

**TABLE 1:** Overview of collagen types and their roles in various tissues.

Collagen type	Where it’s found?	What it does?	References
Collagen I	Connective tissues, Bone, Ligaments, Skin, and Tendon	Provides toughness to skin, bone, and fibrous tissue - Strength Provider	Henriksen and Karsdal (2024), Kanniyappan et al. (2024a), 2024b
Collagen II	Cartilage, Eyes	Handles joint pressure and keeps the cartilage healthy – Shock Absorber.	Lai et al. (2020)
Collagen III	Connective tissues, Organs, Blood vessels, Skin	Provides strength and shape to the tissues - Supporter	Kuivaniemi and Tromp (2019)
Collagen IV	Basement membranes (e.g., Kidneys), Heart valves	Creates a network that supports cells and filters biomolecules – Filter	Bersie-Larson et al. (2020)
Collagen V	Skin, Bone, Cornea	Work along with Type I collagen to provide strength and structure to the tissues – Fibril Regulator.	Wenstrup et al. (2011), Karsdal (2023)
Collagen VI	Connective tissue, muscles	Helps to stay organized and recover tissues – Repair worker.	Di Martino et al. (2023)
Collagen VII	Skin	Holds skin layers together - Anchor	Gretzmeier et al. (2022)
Collagen VIII	Cornea, Blood vessels	Helps the growth of blood vessels and movement of cells- Builder	Mohabeer et al. (2021)
Collagen IX	Cartilage	Keeps cartilage strong by linking collagen to other molecules - Stabilizer	Heilig et al. (2020)
Collagen X	Growth plate in bones	Supports formation of bone and growth during development – Body Builder	Shen (2005), Kamakura et al. (2023)
Collagen XI	Cartilage	Controls the arrangement of collagen fibers in cartilage - Organizer	Sun et al. (2020)
Collagen XII	Tendons, ligaments, and Skin	Strengthens tissues under tension-like ligaments and tendons - Reinforcer	Izu et al. (2021)
Collagen XIII	Skin, Blood vessels	Helps tissues stay attached during stress or movement - Stabilizer	Heikkinen et al. (2020)
Collagen XIV	Tendons, Skin	Works with other collagens to keep fibers organized – Fibril Helper.	Young et al. (2000), Gillesberg et al. (2024)
Collagen XV	Blood vessels	Helps maintain the structure of blood vessels and other tissues - Stabilizer	Muona et al. (2002), Bretaud et al. (2020)
Collagen XVI	Smooth muscle, connective tissue	Helps cells stick to their surroundings – Adhesion Specialist	Eble et al. (2006)
Collagen XVII	Skin (Basement Membrane)	Connects layers of skin, keeping them together - Glue	van Leusden et al. (2001)
Collagen XVIII	Blood vessel walls	Controls growth of blood vessels – Angiogenesis Regulator	Seppinen et al. (2008)
Collagen XIX–XXVIII	Specialized tissues	Works in essential roles of unique tissues – Special Team	Munezane et al. (2019)

Collagens’ essential involvement in tissue specificity and homeostasis is reflected in their structural and functional diversity. The clinical impact of impaired collagen integrity is significant, as mutations in collagen genes can lead to serious connective tissue disorders. For example, mutations in COL1A1-COL1A2 cause osteogenesis imperfecta, mutations in COL4A3–COL4A5 result in Alport syndrome, and COL3A1 mutation leads to vascular Ehlers-Danlos syndrome (O’Connell, 2014).

## 3 Collagen-related disorders

Collagen disorder impacts different body parts, leading to discomfort, impairment, and even fatal consequences due to weakened tissues and compromised functions when the synthesis, assembly, or degradation of collagen is disrupted. As discussed in the previous section, the ratio between the two primary forms of collagen-collagen I and collagen III- is crucial to mitigate the risks of these disorders. Firm and dense collagen I offers structural support to tissues like bones, ligaments, and tendons. Collagen III, which is more elastic and flexible, is found in tissues that need to stretch and recoil, such as blood vessels, skin, lungs, and intestines. A proper ratio of the two collagen types in a healthy tissue warrants a balance between the tissue’s strength and flexibility (Table 2). A disruption of this equilibrium can result in an array of pathophysiological conditions, such as bone disorders, chronic wounds, periodontal disease, and pelvic organ prolapse (POP), among others (Chi et al., 2022).

**TABLE 2:** The ratio of Collagen I and Collagen III (collagen I/III) in various tissues.

Tissue type	Collagen I/III	References
Skin	2.1–3.1	Li et al. (2021b)
Human heart	0.3–0.6	Wittig and Szulcek (2021)
Blood vessels	2–3:1	Shabani et al. (2024)
Tendons & ligaments	38:1	Birch et al. (2013), Tresoldi et al. (2013)
Bone	Collagen I: >90%; Collagen III: <10%	Amirrah et al. (2022)
Intestine	2:1	Brown et al. (2017)
Liver	1:1	Cequera and García de León Méndez (2014)
Uterus (Non-Pregnant)	2–3:1	Yellon (2020)
Scar tissue	5:1	Li et al. (2021b)

In this review, we highlight several key collagen-related pathological conditions, such as POP, osteoarthritis (OA), diabetic wound healing, osteoporosis, and periodontal disease. These conditions are clinically prevalent and physiologically diverse. They represent significant health challenges and considerable physical and economic burdens. We aim to elucidate these conditions and divulge treatment options by looking into the underlying mechanisms associated with the role of collagen imbalance. The insights offer the prospects of improving clinical outcomes and quality of life for affected individuals.

### 3.1 Pelvic organ prolapse

**FIGURE 3:**
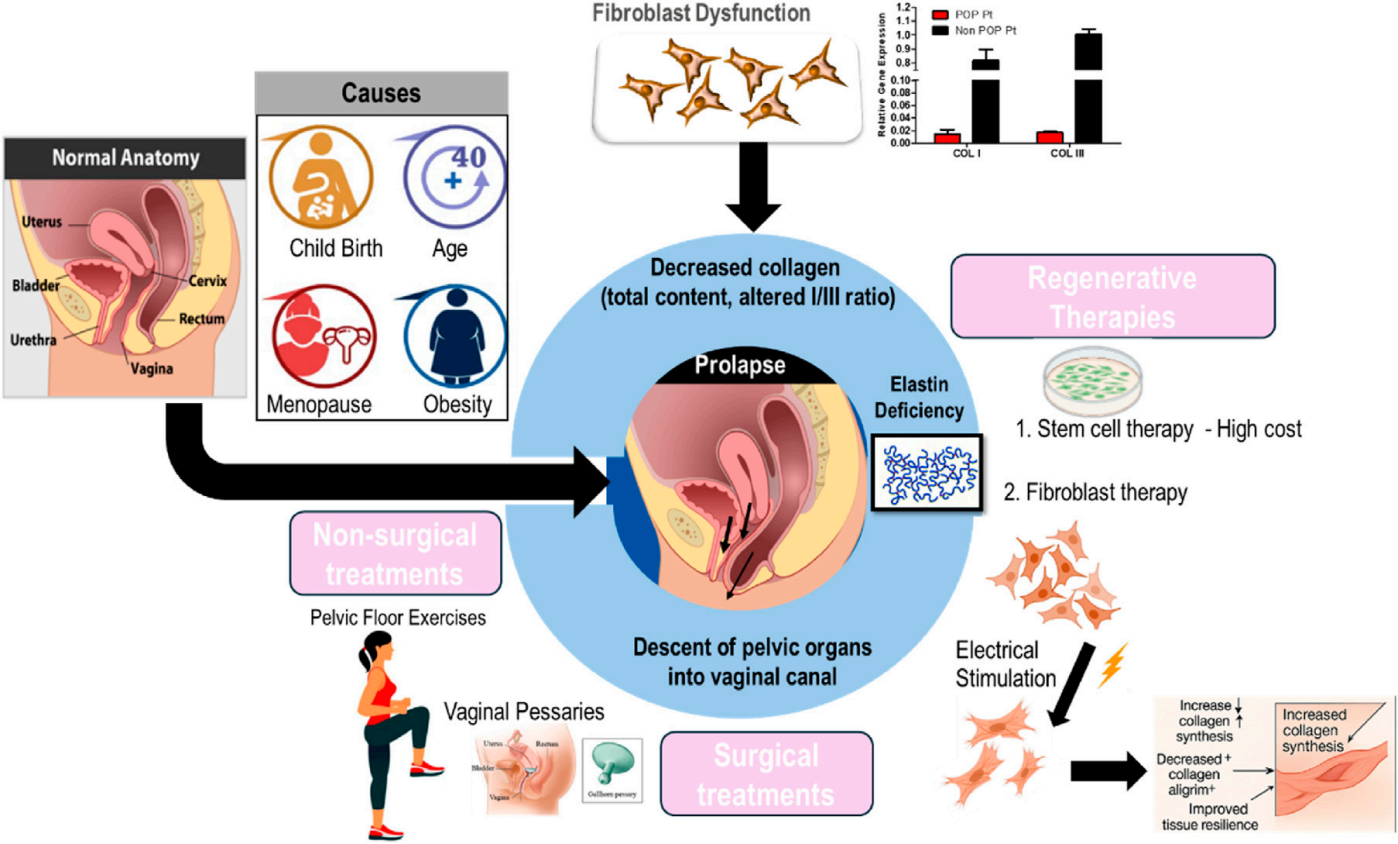
Overview of the pathophysiology of pelvic organ prolapse and the current clinical treatment modalities.

Globally, approximately 40% of women are projected to encounter POP (Carroll et al., 2022). It is a condition characterized by the weakening of pelvic floor muscles and connective tissues, resulting in the descent of pelvic organs into the vaginal canal. POP affects women of various ages and can lead to pelvic pressure, vaginal bulge, urinary and bowel issues, and sexual dysfunction, particularly in older adults. Growing evidence links connective tissue abnormalities to the development of POP (Ferreira et al., 2020; Wang B. et al., 2022). POP results from compromised pelvic floor support, primarily due to alterations in connective tissue composition and structure. The pelvic floor’s support matrix is composed of collagen (mainly collagen I and collagen III), elastin, and smooth muscle cells. In POP, there is a documented reduction in total collagen content, an increased collagen I/III ratio (Li et al., 2021b; Chi et al., 2022), increased expression of MMPs, changes in collagen cross-linking, which collectively reduce tissue resilience and mechanical strength and abnormalities in the nanoscopic to microscopic structure of fibrillar collagen within the vaginal wall connective tissues of POP patients were identified in our previous studies (Sridharan et al., 2012; Kim et al., 2016). Elastin deficiency further impairs tissue elasticity, while smooth muscle dysfunction contributes to loss of active support (Jameson et al., 2020). Our previous studies demonstrated that fibroblasts isolated from prolapsed tissues display an altered response to mechanical stimulation and a diminished ability to synthesize collagen (Persu et al., 2011; Wang X. et al., 2022) (Figure 3).

POP is usually diagnosed through a pelvic examination. This examination involves the evaluation of the patient’s pelvic organs while they exert abdominal pressure and recline in a supine position (Persu et al., 2011; Chi et al., 2022). POP Management is individualized, considering both the severity of the prolapse and the patient’s preference. Mild cases are typically managed conservatively through lifestyle modifications, pelvic floor exercises (such as Kegels), and the use of pessary devices. In contrast, more severe or persistent prolapse may require surgical intervention, ranging from traditional open procedures to minimally invasive techniques, to restore normal pelvic anatomy. Ultimately, optimal care requires a personalized approach, guided by thorough assessment and professional expertise (Chung and Kim, 2018).

Despite the range of available treatments—including surgical repair and pessary use—high recurrence rates and various complications remain significant challenges (Kelly et al., 2016; Wu et al., 2017). Pelvic floor rehabilitation training often falls short of providing satisfactory results. Hence, there is a pressing need for novel, safer, and more effective treatment modalities. Stem cell therapy, particularly utilizing bone marrow mesenchymal stem cells (MSCs), has garnered attention due to their self-renewal and differentiation capabilities. However, challenges such as prolonged culture periods, limited tissue availability, and high costs hinder widespread adoption (Herberts et al., 2011; Wang X. et al., 2022). Additional barriers to care for pelvic floor disorders include underreporting of symptoms, misdiagnosis, and systemic healthcare delays—issues that are especially common among older women. In light of these challenges and the limitations of current treatments, several regenerative approaches are now under investigation as potential solutions (Yuk et al., 2018; Wang X. et al., 2022; Cross et al., 2023). Fibroblasts offer a promising alternative. They are abundant, easily accessible, and amenable to lab cultivation and expansion. Moreover, utilizing autologous fibroblasts presents a straightforward and cost-efficient solution, as these cells are proficient in producing collagen and other essential extracellular matrix proteins (Chi and Wang, 2018; Chi et al., 2019).

In our prior research, it was demonstrated that stimulated fibroblast cells can produce an increased amount of collagen, which holds promise for their use in the repair of chronic wounds and POP (Chi and Wang, 2018; Chi et al., 2019). Building on these findings, electrical stimulation effectively corrects fibroblast dysfunction and increases collagen synthesis, supporting improved pelvic floor integrity. This enhanced collagen production is accompanied by a decreased collagen alignment index, suggesting improved tissue biomechanics and resilience (Rathnayake et al., 2022). Our *in vivo* studies further validated these findings, underscoring the therapeutic promise of ES in addressing collagen-related disorders. By both enhancing collagen deposition and modulating its structural alignment, ES may be pivotal in restoring the biomechanical integrity of pelvic tissues, potentially aiding in the prevention or mitigation of POP and similar conditions. The reproducibility of our results across *in vitro* and *in vivo* models highlights the reliability of ES as a strategy for activating fibroblast function and promoting collagen synthesis.

### 3.2 Ovarian cancer and ECM remodeling

**FIGURE 4:**
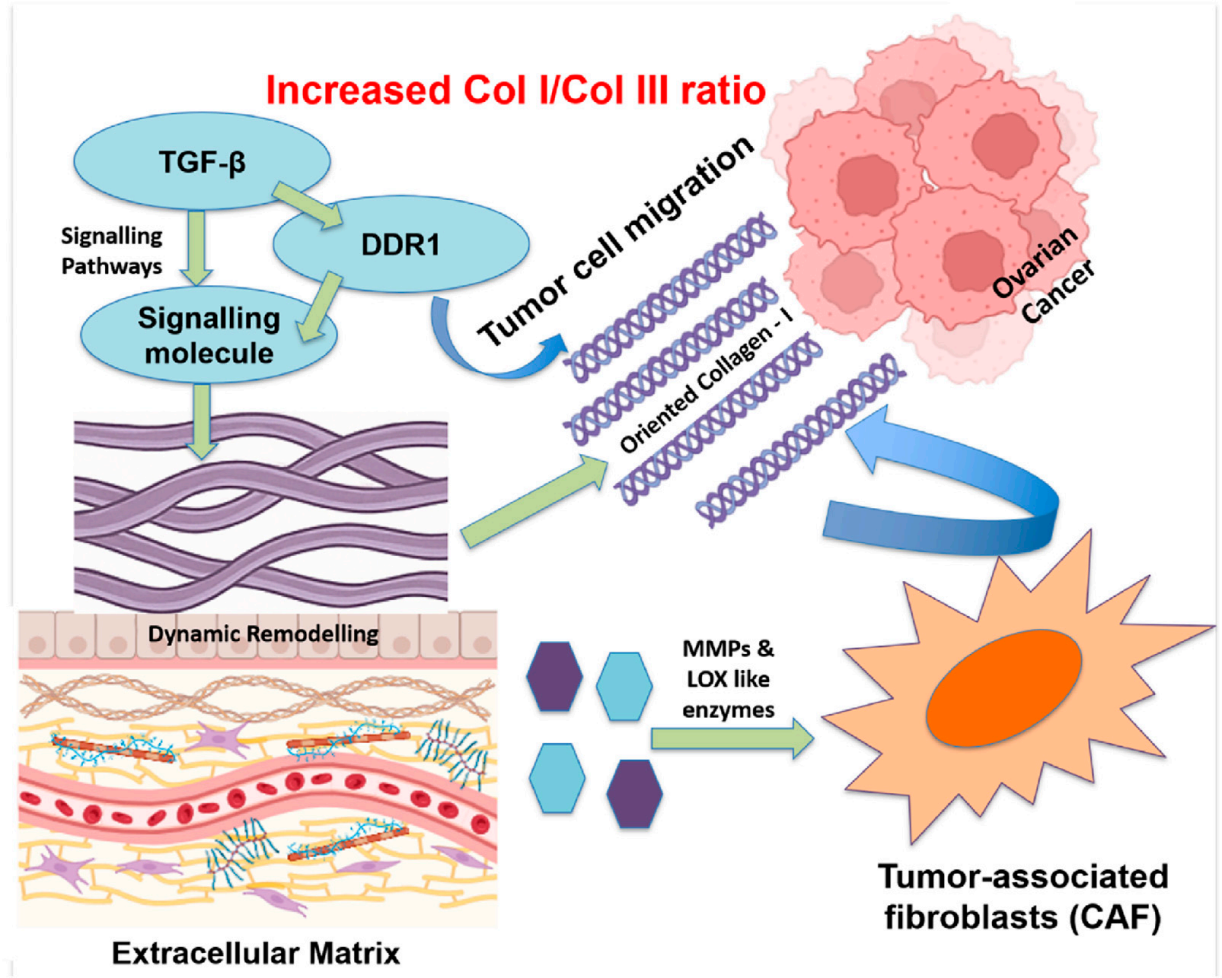
Realignment of Collagen fibers in the ECM of ovarian tumors to assist cancer spreading. Specialized groups of cells, such as tumor-associated fibroblasts, release enzymes that break down and rebuild the ECM. Signals from molecules, e.g., TGF-β and DDR1, facilitate collagen I alignment, forming “tracks” that guide cancer cells to migrate. A higher collagen I/III ratio in the ECM creates a stiffer environment which supports tumor growth and invasion.

Ovarian cancer (OC) is the most lethal gynecologic malignancy, often diagnosed at an advanced stage because of its ambiguous early symptoms. The development and spread of tumors are significantly influenced by ECM remodeling, especially collagen realignment. Cancer cells and tumor-associated fibroblasts (CAFs) secrete MMPs and lysyl oxidase-like enzymes. These enzymes alter the extracellular matrix by changing its biochemical composition, structure, and stiffness. In the ovarian cancer microenvironment, there is a significant increase of the collagen I/III ratio. This microstructural shift leads to enhanced stiffness and realignment of collagen fibers, forming linear and oriented fibrillar structures that are aligned perpendicular to the tumor margin, creating “highways” that facilitate directional migration of cancer cells through contact guidance-mediated mechanotransduction (Erez et al., 2010; Li et al., 2021a; Thorseth et al., 2022) as represented in Figure 4.

Signaling pathways, mediated by factors such as transforming growth factor beta (TGF-β), integrins, and discoidin domain receptor 1 (DDR1), control this remodeling. It is observed that DDR1 is involved in collagen alignment mediation and metastasis promotion in ovarian cancer (Quan et al., 2011; Wang S. et al., 2022). A different investigation conducted by *Pickup et al.* brought attention to the function of TGF-β signaling in controlling collagen’s arrangement and accelerating tumor growth of tumors (Pickup et al., 2013). The potential of blocking ECM remodeling by DDR1 antagonists, anti-TGF-β antibodies, and MMP inhibitors (like batimastat) is being studied as a treatment option for tumor metastases (Nam et al., 2008; Liu and Khalil, 2017; Winer et al., 2018; Yang et al., 2020; Wu et al., 2024). On the other hand, breaking the tumor-ECM interaction by anti-fibrotic drugs and CAF-targeting treatments is expected to lessen invasiveness and enhance the delivery of chemotherapy (Jiang et al., 2022).

Multiple studies have underscored the clinical consequences of collagen realignment for tumor invasion. For instance, it was shown by *Natarajan et al.* that the collagen fibers in the ECM of ovarian tumors are oriented perpendicular to the tumor margin, offering structural support for the invasion of the tumor (Natarajan et al., 2019). According to *Levental et al.,* collagen alignment facilitates ovarian cancer cell motility and improves its capacity to permeate the extracellular matrix (Levental et al., 2009). The significance of ECM architecture in cancer treatment has been highlighted by studies employing 3D tumor models, demonstrating that altering collagen alignment hinders tumor invasion (Staneva et al., 2018; Alkmin et al., 2022).

The arrangement of collagen in healthy tissues is frequently more loosely arranged or randomly oriented. Conversely, in malignancies, an increased collagen I/III ratio and collagen I enrichment led to excessive fiber alignment, which in turn enhances matrix stiffness and facilitates directional cell migration. Collagen realignment for the tumor invasion within the microenvironment of ovarian tumors is intricate and involves multiple components. Comprehending the interplay between these components and the collagen I/III ratio is crucial in formulating novel treatment approaches that aim to target the extracellular matrix, hence impeding tumor advancement and spread.

### 3.3 Diabetic wound healing

**FIGURE 5:**
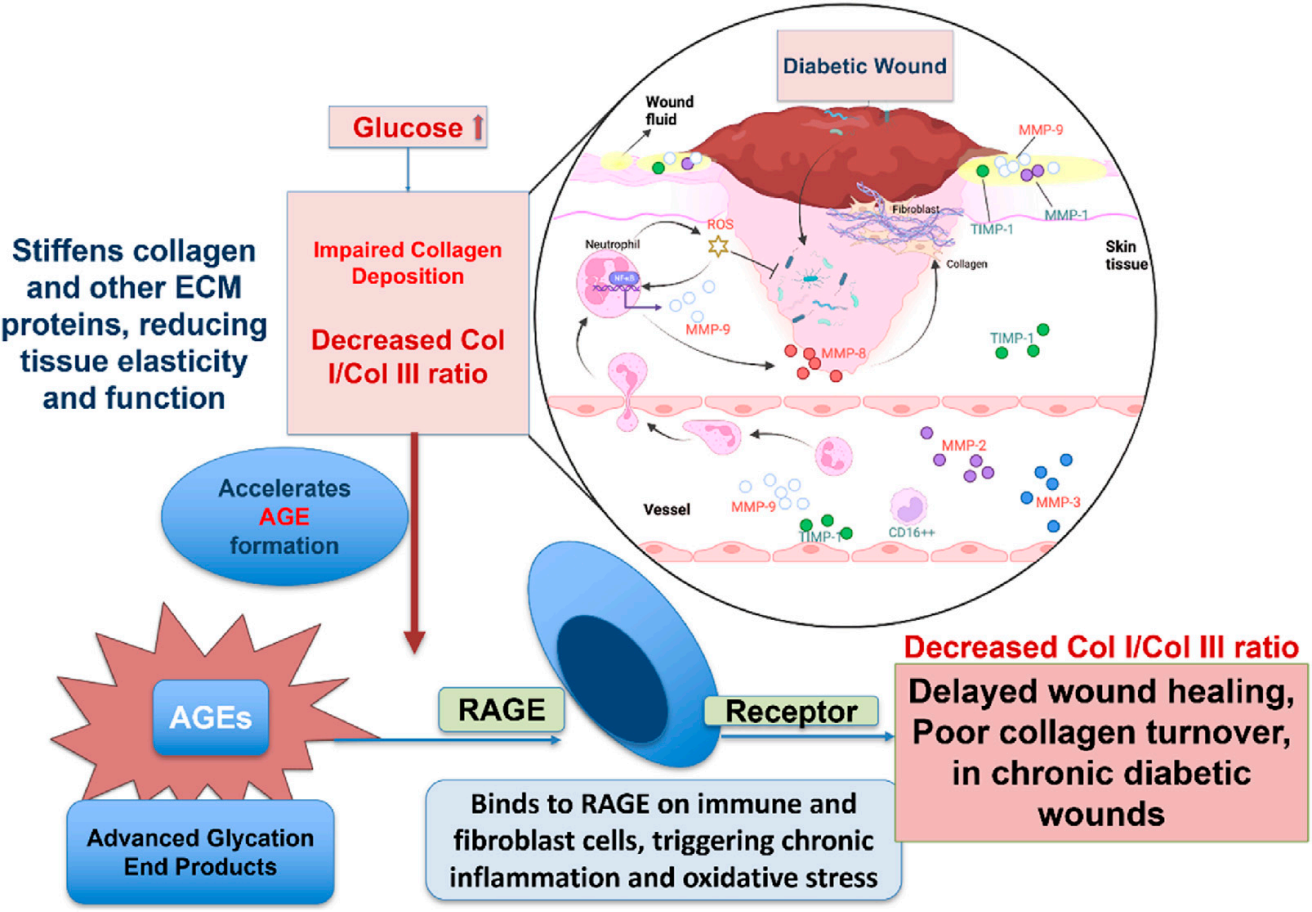
Diagram illustrating how high glucose levels in diabetes disrupt wound healing by impairing collagen deposition. Excess glucose promotes AGE formation, which stiffens tissue and triggers chronic inflammation through RAGE receptors. This leads to a decreased collagen I/III ratio, resulting in delayed healing and poor tissue repair in diabetic wounds.

Chronic wounds represent a major complication of diabetes mellitus, significantly increasing patient morbidity and healthcare expenditure. These lesions, which frequently manifest as diabetic foot ulcers, are distinguished by inadequate angiogenesis, ongoing inflammation, and abnormal ECM remodeling as shown in Figure 5. Defective collagen deposition constitutes a hallmark of impaired diabetic wound healing (Südy et al., 2021).

Collagen molecules of different types are deposited dynamically and sequentially during proper wound healing. For instance, collagen III first lays the groundwork for collagen I to replace it later to create a robust, mature matrix. This development has stopped or is significantly delayed in diabetes patients. An elevated glucose level limits the fibroblasts’ capacities to generate collagen, proliferation, and mobility. Moreover, hyperglycemia promotes the formation of advanced glycation end-products (AGEs), which cross-link collagen fibers, increasing the matrix stiffness and impairing its biological functionality (Guillon et al., 2021). AGEs perpetuate inflammation and oxidative stress by binding to their specific receptors (Receptor for AGEs -RAGE) on immune cells and fibroblasts (Chuah et al., 2013). Chronic wounds with an excess of immature collagen III and a shortage of collagen I result from these causes.

Therapeutic strategies for diabetic wounds increasingly target the ECM (Holl et al., 2021). Collagen-based wound dressings offer structural scaffolding and create an optimal microenvironment for angiogenesis and cellular infiltration. Incorporation of bioactive compounds, such as growth factors (vascular endothelial growth factor (VEGF), platelet-derived growth factor (PDGF)), oxygen-releasing compounds, or antimicrobial peptides, effectively enhances healing responses. Autologous platelet-rich plasma and exosomes produced from stem cells have demonstrated efficacy in modulating the wound microenvironment and promoting collagen synthesis (Huang et al., 2025). Non-invasive modalities, such as electrical stimulation and photo-biomodulation, are gaining recognition as effective approaches to upregulate collagen I gene expression and improve wound healing outcomes in diabetic patients (Xu et al., 2022; Oyebode et al., 2023; Mgwenya et al., 2025).

### 3.4 Osteoporosis

**FIGURE 6:**
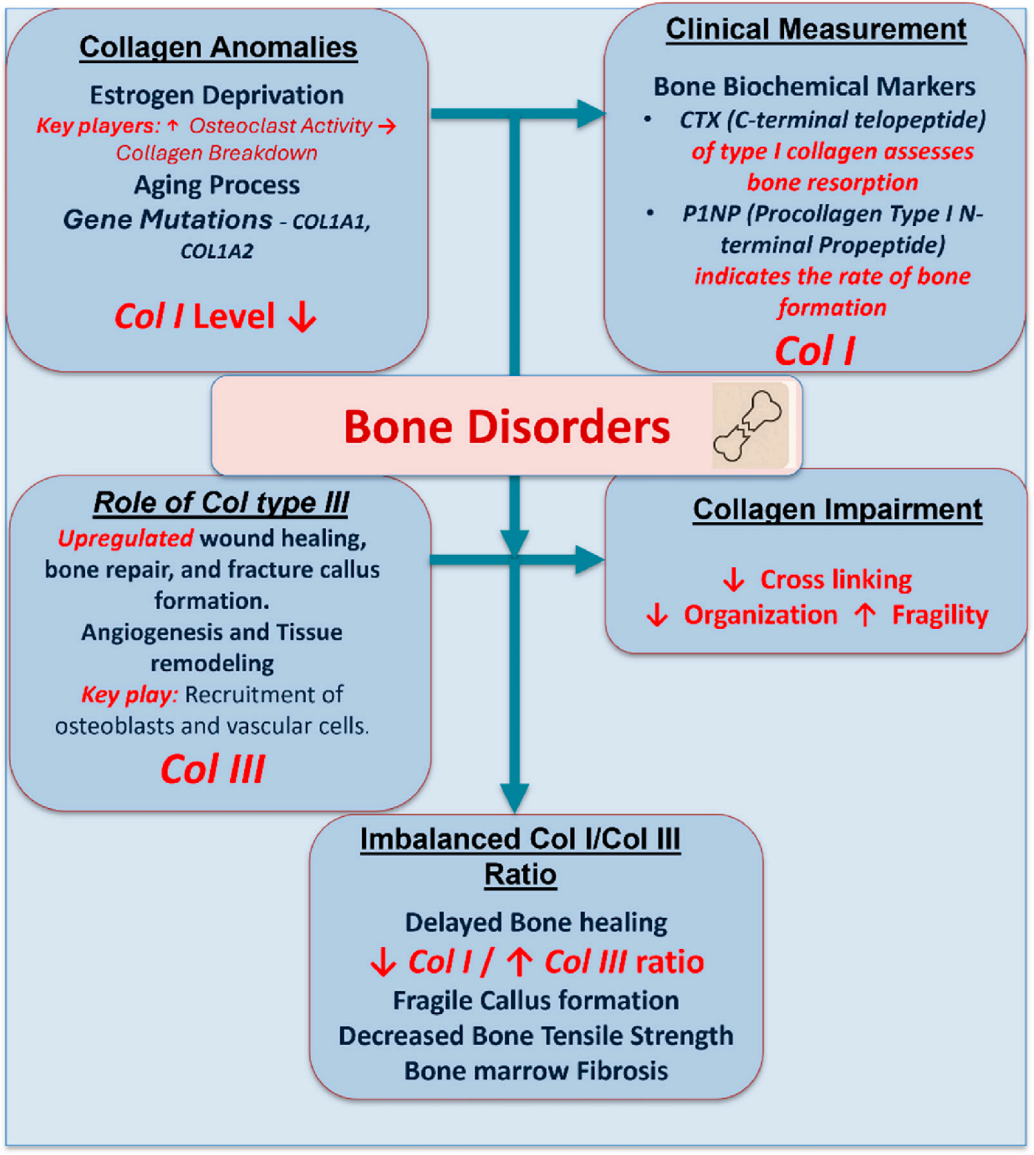
A schematic demonstration showing the role of collagen I and collagen III in bone disorders. Estrogen deprivation, aging, and mutations downregulate the collagen I level and upregulate the collagen III level. This imbalance impairs bone quality, healing, and tensile strength, contributing to bone fragility, marrow fibrosis, and delayed callus formation.

Reduced bone mass, microarchitectural degradation, and an elevated risk of fracture are the hallmarks of osteoporosis, a systemic skeletal condition (Wen et al., 2012; Mukherjee and Das, 2024). Collagen I is a significant portion of the bone matrix and plays an essential role in the bone matrix’s tensile strength to withstand fractures (Mäkitie et al., 2019; Kanniyappan et al., 2020; Kanniyappan et al., 2021; Kanniyappan et al., 2024a). The mechanical integrity of the bone is compromised in osteoporosis due to disruptions in collagen synthesis, cross-linking, and organization. Collagen abnormalities in bone disorders result from both disease-specific mechanisms and natural aging processes that impair the quality of bone, mechanical properties, and healing mechanisms, leading to bone marrow fibrosis and bone fragility (Figure 6). For example, estrogen deprivation in postmenopausal women increases osteoclast activity and promotes collagen breakdown. Osteogenesis imperfecta or an increased fracture risk can also be caused by mutations in collagen genes COL1A1 and COL1A2 (Sun et al., 2024; Subramanian et al., 2025).

In clinical practice, collagen turnover is measured using bone resorption markers such as C-terminal telopeptide of collagen I (CTX) and bone formation markers such as procollagen type I N-terminal propeptide (P1NP) (Uitterlinden et al., 1998). Therapeutic approaches aim to rebalance bone remodeling. Bisphosphonates and denosumab are examples of anti-resorptive drugs that prevent osteoclast-mediated collagen deterioration. Teriparatide and romosozumab are anabolic therapies that promote osteoblast function and collagen I synthesis. Tissue-engineering techniques are being developed to use collagen scaffolds in conjunction with mesenchymal stem cells, bioactive glass, or hydroxyapatite to rebuild osteoporotic bone. These methods aim to improve collagen’s structure and quality inside the bone matrix, in addition to restoring bone density (Zhang et al., 2017; Acri et al., 2021).

### 3.5 Periodontal disease

**FIGURE 7:**
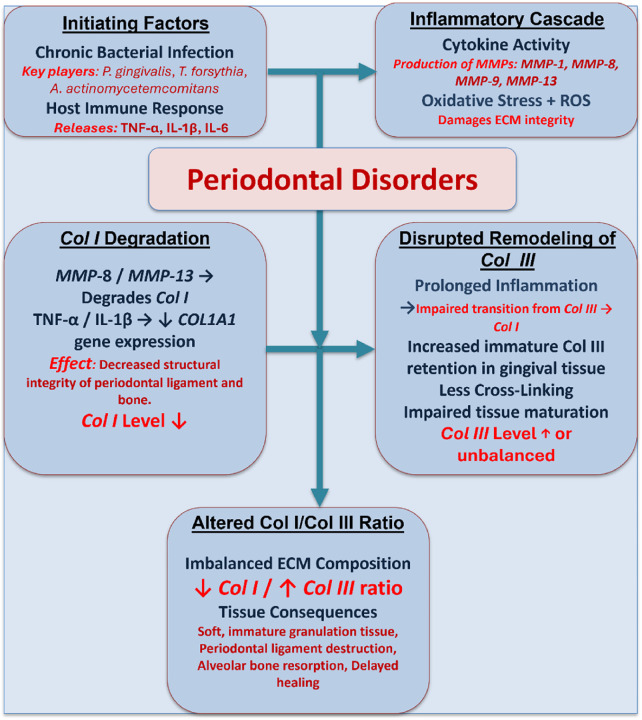
A schematic diagram illustrating the pathophysiological mechanisms that regulate the collagen I/III ratio in periodontal disorders. Chronic infection and inflammation trigger MMP production and oxidative stress, leading to the degradation of collagen I and disrupted remodeling of collagen III, which results in an imbalanced ECM and impaired periodontal healing.

Chronic bacterial infections and dysregulated host immune responses are the main causes of periodontal disease, an inflammatory disorder that impair the teeth’s supporting tissues (Kwon et al., 2021). Both microbial enzymes and host-derived proteases, e.g., MMPs, significantly degrade the ECM of periodontal tissues, which is rich in collagen I and collagen III (Isler et al., 2022). Prolonged inflammation causes the periodontal ligament to break down, the gingival collagen to degrade, and the alveolar bone to resorb as periodontitis progresses. MMP-8 and MMP-13 are essential for breaking down collagen I in periodontal tissues. In the normal periodontal ligament, collagen I represents the major structural component of the fibrillar collagen network with a collagen I/III ratio of 4:1. Nevertheless, periodontitis significantly disrupts this ratio through selective collagen I degradation while maintaining or enhancing collagen III synthesis, resulting in a pathological shift in collagen composition (Kaku and Yamauchi, 2014; Li et al., 2019). At the same time, pro-inflammatory cytokines, including tumor necrosis factor-alpha (TNF-α) and interleukin-1 beta (IL-1β), exacerbate the degradative microenvironment by suppressing collagen synthesis while upregulating MMP expression (Franco et al., 2017; Luchian et al., 2022). The pathophysiological mechanisms that regulate the collagen I/III ratio in periodontal disorders are represented in Figure 7.

**FIGURE 8:**
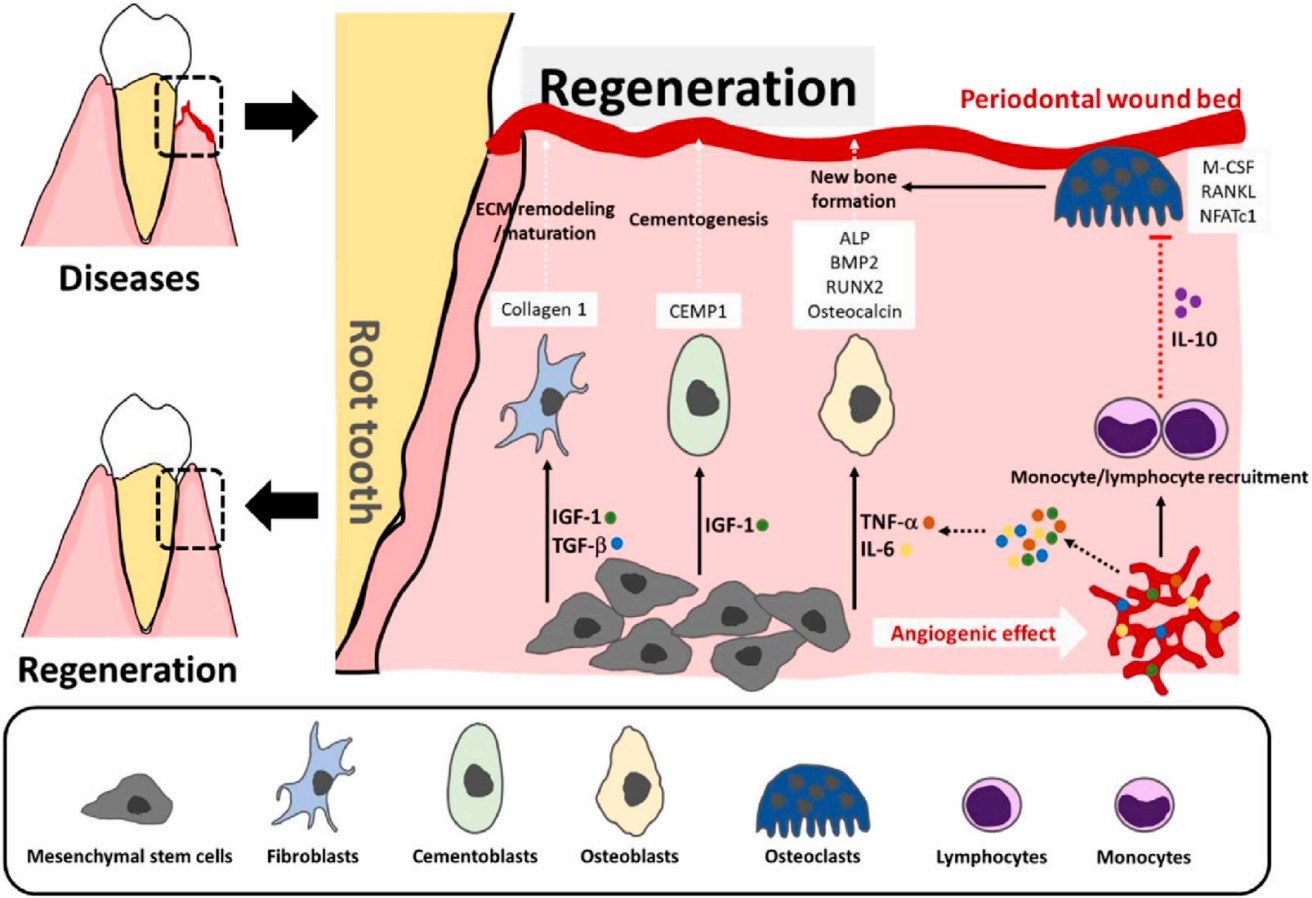
Stem cell-based therapies for periodontal tissue repair. At sites of periodontal damage, MSCs help form new blood vessels and release signaling factors, such as TNF-α, IL-6, IGF-1, and TGF-β, which attract immune cells and guide MSC differentiation to fibroblasts, cementoblasts, and osteoblasts—supporting tissue repair and bone growth. The immune cells release IL-10 to help prevent bone breakdown and stabilize the new bone (Binlateh et al., 2022).

Current therapeutic techniques include surgical procedures, antimicrobial medication, and mechanical debridement. Regenerative periodontal therapy employs collagen-based membranes for guided tissue regeneration, frequently enhanced with bone grafts or enamel matrix derivatives to promote selective regeneration of periodontal ligament, cementum, and alveolar bone (Takallu et al., 2024). Advancement in biomaterial science enables the creation of cross-linked collagen scaffolds that support osteoblast and fibroblast activity and show extended stability (Wen et al., 2024). Additionally, emerging therapeutic modalities including stem cell-based therapies and controlled growth factor delivery methods are under investigation to promote collagen synthesis and comprehensive periodontal tissue repair (Figure 8) (Binlateh et al., 2022).

## 4 Emerging therapeutic strategies

Though we mentioned the treatment strategies in each disorder specifically, in this section, we emphasize the additional treatment strategies and emerging technologies for the effective repair and regeneration of collagen matrices**.**

### 4.1 Collagen-based biomaterials

Collagen-based biomaterials have emerged as significant contributors to regenerative medicine by creating scaffolds mimicking the native ECM while promoting cellular adhesion, proliferation, and differentiation. These materials have found versatile applications across multiple medical fields, including cardiovascular repair, orthopedic reconstruction, wound healing, and cosmetic procedures. While native collagen offers inherent advantages such as biocompatibility and biodegradability, its clinical utility is often limited by suboptimal mechanical properties and susceptibility to enzymatic degradation. Frequently, chemical cross-linking strategies are employed to address these limitations.

The development of hybrid biomaterial systems represents a major advancement in overcoming collagen’s inherent limitations. By combining collagen with synthetic polymers such as poly (lactic-co-glycolic acid) (PLGA), polycaprolactone (PCL), or polyethylene glycol (PEG), researchers can create composite scaffolds with tunable degradation rates, mechanical characteristics, and bioactivity that are tailored to specific clinical applications (Fernandes-Cunha et al., 2020). Further enhancement of scaffold functionality is achieved through the strategic incorporation of bioactive nanoparticles. Nanoparticles such as graphene oxide, bioactive glass, or hydroxyapatite can be integrated into collagen matrices to improve osteoconductivity and bioactivity. Advanced manufacturing techniques have revolutionized the fabrication of collagen-based scaffolds, enabling unprecedented control over scaffold architecture and spatial distribution of bioactive components throughout the scaffold structure. Complementary manufacturing approaches, including electrospinning and freeze-drying techniques, enable the creation of nanofibrous collagen matrices that closely resemble native tissue structures (Coolen et al., 2019; Marques et al., 2019; Jonidi Shariatzadeh et al., 2025).

### 4.2 Stem cell therapy

**FIGURE 9:**
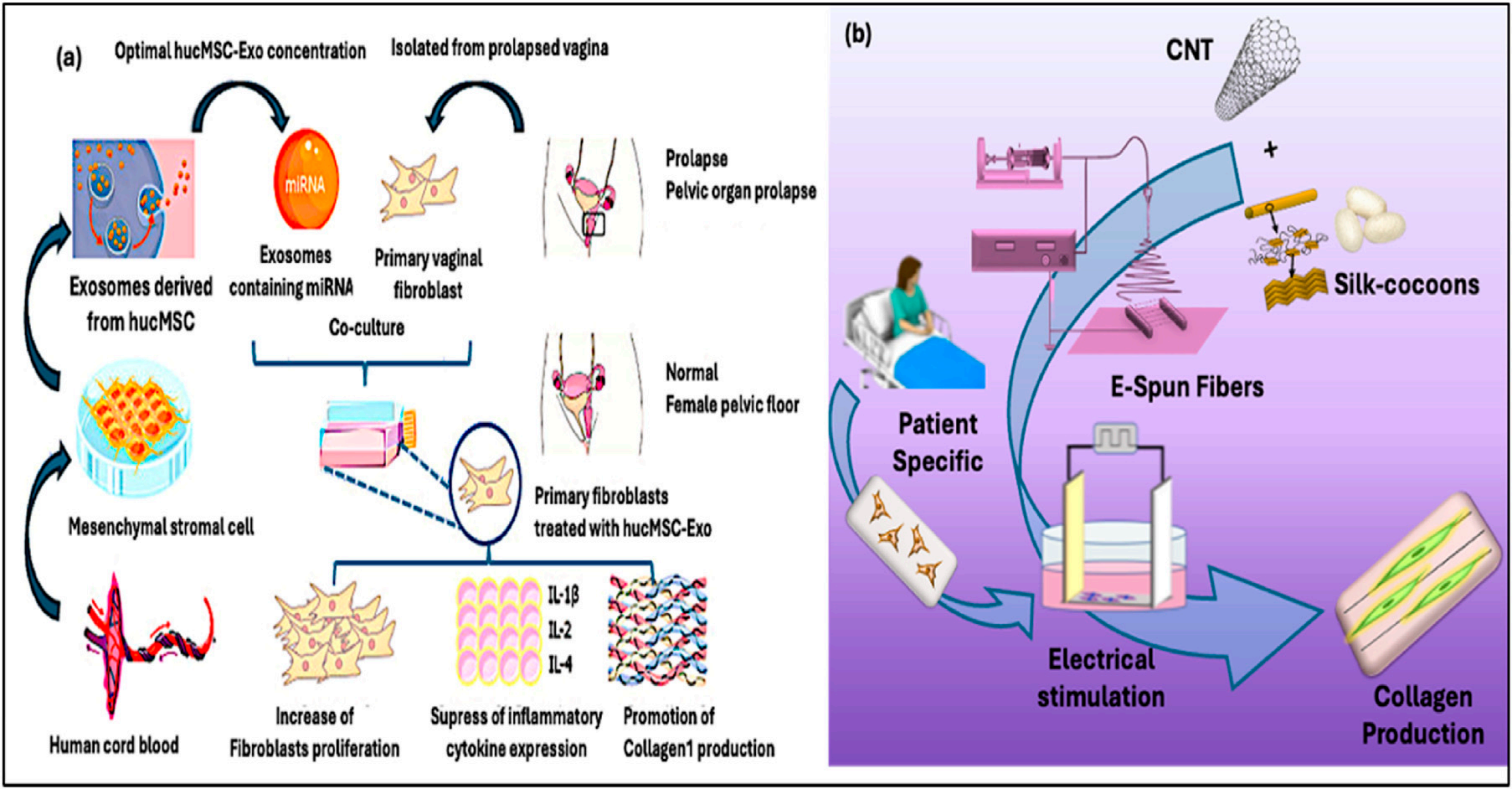
**(a)** Exosomes derived from human umbilical MSCs promote the proliferation and collagen I production of fibroblasts through the miRNA (Xu et al., 2024). **(b)** Electrospun silk fibroin-CNT fibers are mechanically strong, electrically conductive and well aligned with structure mimicking the ECM of connective tissues. The composite fibers effectively mediated the electrical stimulation of fibroblasts derived from POP patients and boosted the production of collagen I and collagen III at a ratio favorable for rejuvenation (Rathnayake et al., 2022).

Since stem cells are regenerative and immunomodulatory, stem cell–based therapies hold great promise for treating collagen-associated diseases. MSCs obtained from tooth pulp, bone marrow, adipose tissue, or the umbilical cord can develop into osteoblasts and fibroblasts, aiding tissue remodeling and collagen production. MSCs seeded onto biomaterials have shown to effectively remodel the collagen matrix with improved collagen I/III ratio and increased biomechanical strength to manage pelvic organ prolapse (Zhang et al., 2021). MSCs also exerted paracrine effects on diabetic wounds that promote endogenous fibroblasts and decrease inflammation. Moreover, it has been demonstrated that proteins and microRNAs carried by exosomes and extracellular vesicles produced from stem cells increase the expression of the collagen gene. For instance, *Xu et al.* showed that the human umbilical mesenchymal stem cell (hucMSC)-derived exosomes contain advantageous microRNAs that may aid in pelvic tissue repair. Upon the introduction of these exosomes to primary vaginal fibroblasts derived from patients with POP, they were found to facilitate the reduction of inflammation through the downregulation of critical cytokines (e.g., IL-1β, IL-2, IL-4), promote fibroblast proliferation, and significantly enhance collagen I synthesis—essential processes for the restoration of a healthy pelvic floor (Figure 9a) (Xu et al., 2024).

Despite their therapeutic potential, challenges such as immunogenicity, variability in stem cell quality and tumorigenic risk persist. To address these limitations, induced pluripotent stem cells (iPSCs) and genetically engineered MSCs are under investigation. Furthermore, bioengineering approaches—including hydrogel encapsulation, hypoxia preconditioning, and 3D biomimetic scaffolds—are being employed to improve stem cell viability, retention, and functionality *in vivo* (Sridharan et al., 2009; Sridharan et al., 2013; Li et al., 2014; Zhu et al., 2014; Zhu et al., 2015; Zhu et al., 2017; Kim et al., 2015).

### 4.3 External stimulation

Non-invasive physical techniques, such as electrical stimulation (ES), photo-biomodulation (PBM), and mechanical loading, have emerged as adjunctive therapeutic interventions for modulating tissue regeneration and collagen biosynthesis (Park et al., 2015; Woo, 2024). ES represents the most extensively studied physical modality for collagen enhancement through coordinated activation of calcium influx, mitogen-activated protein kinase pathway (MAPK) pathways, and TGF-β signaling in fibroblasts that collectively upregulate COL1A1 and COL1A2 gene transcription (Peng et al., 2025). Clinical applications span from pelvic floor rehabilitation, post-surgical recovery, to wound management. Our recent studies demonstrated that aligned silk fibroin-carbon nanotube scaffolds effectively mediated the electrical stimulation of fibroblasts of POP patients to promote collagen synthesis at a desirable collagen I/III ratio favorable for pelvic tissue rejuvenation and repair (Figure 9b) (Chi and Wang, 2018; Chi et al., 2019; Rathnayake et al., 2022).

PBM uses red or near-infrared light to increase cellular adenosine triphosphate (ATP) synthesis and mitochondrial function. This photo-stimulation lowers inflammation and oxidative stress while increasing fibroblast migration, proliferation, and collagen deposition (Hamblin, 2017). Clinical research shows that PBM increases the tensile strength of repaired tissues, speeds up wound closure, and improves scar quality (Anders et al., 2015). Mechanical stimulation, especially in synthetic tissues, activates mechanotransducive pathways via the integrin-mediated signal cascades to upregulate collagen gene expression. Simulating physiological stress, it promotes ECM formation and functional integration (Dasgupta and McCollum, 2019).

Current research efforts focus on developing multimodal therapeutic platforms that integrate physical stimulation techniques with advanced biomaterial scaffolds and stem cell therapies to address collagen deficiency. The clinical utility is further expanded by sophisticated delivery methods incorporating smart hydrogels and wearable electronics. For example, conductive hydrogel patches embedded with electrodes can provide localized ES therapy while simultaneously delivering MSCs or growth factors directly to target tissues. These integrated platforms represent a paradigm shift toward personalized, multi-target therapeutic strategies that address the complex pathophysiology underlying collagen-related disorders.

### 4.4 Gene and molecular therapies

**FIGURE 10:**
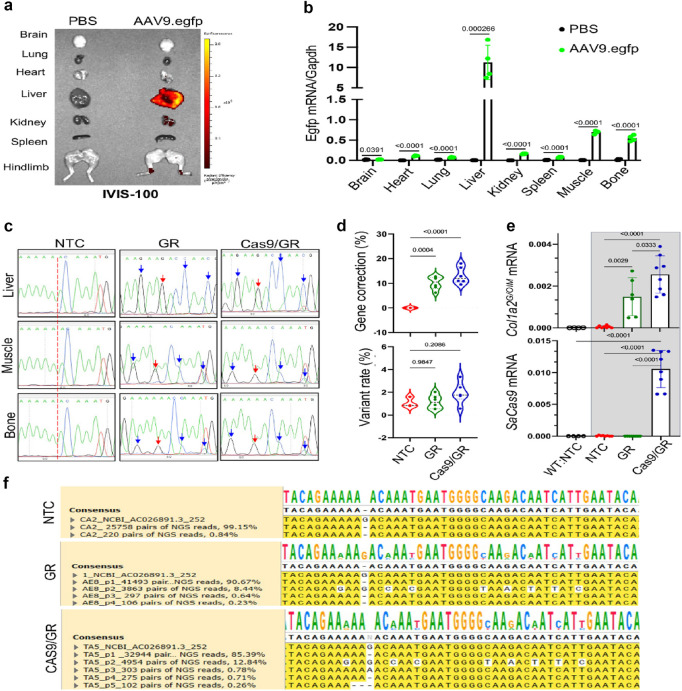
An AAV-based gene editing approach for collagen I mutation to treat osteogenesis imperfecta in mice. **(a)** Demonstration of the frameshift mutation in the COL1A2 gene of OIM mice. **(b)** Repairing template sequence of the pro-a2 C-terminal domain of the propeptide. **(c–e)** Gene correction of the COL1A2 gene mutation in IOM osteoblasts was achieved using rAAV9-mediated delivery of GeneRide and Cas9/GR, analyzed through Sanger and NGS analysis. **(f)** mRNA levels of corrected genes (Yang et al., 2020).

Gene and molecular therapies focus on the molecular causes of collagen disorders to develop innovative treatment options (Figure 10). CRISPR-Cas9 and related nuclease systems have demonstrated potential for correcting pathogenic variants in critical collagen genes. While proof-of-concept studies have shown successful *in vitro* correction of disease-causing mutations associated with osteogenesis imperfecta and classical Ehlers-Danlos syndrome (Aoki, 2024), significant challenges remain regarding delivery efficiency, off-target effects, and long-term safety for clinical applications (Jung et al., 2021).

Post-transcriptional regulation offers alternative strategies for modulating collagen homeostasis without permanent genomic alterations. Small interfering RNAs (siRNAs) and antisense oligonucleotides (ASOs) have shown efficacy in silencing hyperactive genes encoding matrix metalloproteinases and cathepsins, thereby reducing pathological ECM turnover (Kole et al., 2012). MicroRNA-based therapeutics target key regulatory networks controlling collagen biosynthesis. Specifically, microRNA-29 family members function as negative regulators of multiple collagen genes (COL1A1, COL3A1, COL5A1), while microRNA-21 and microRNA-146a modulate TGF-β signaling and inflammatory responses that influence fibrotic remodeling. Therapeutic strategies employing microRNA mimics or antagomirs are under investigation for conditions ranging from pulmonary fibrosis to hypertrophic scarring (Wang et al., 2012).

Molecular inhibitors targeting the TGF-β/Smad signaling axis represent the most clinically advanced molecular therapies for fibrotic collagen disorders. The activin receptor-like kinase 5 (ALK5) inhibitor galunisertib and the antifibrotic agent pirfenidone have demonstrated efficacy in reducing collagen deposition and improving functional outcomes in Phase II/III clinical trials for idiopathic pulmonary fibrosis, with ongoing investigations in hepatic and renal fibrosis (Herbertz et al., 2015; Antar et al., 2022). These agents may have broader applications in treating pathological collagen accumulation across multiple organ systems.

The clinical translation of gene and molecular therapies faces substantial delivery obstacles, including poor tissue penetration, rapid clearance, and potential immunogenicity. Researchers are investigating hydrogel-based carriers, liposomes, and nanoparticle-based systems to increase targeted distribution, bioavailability, and cellular uptake while reducing off-target effects. Emerging approaches combine these delivery systems with biomaterial scaffolds and cell-based therapies to create integrated treatment platforms that may enhance therapeutic efficacy while minimizing systemic exposure and associated risks.

## 5 Future directions

**FIGURE 11:**
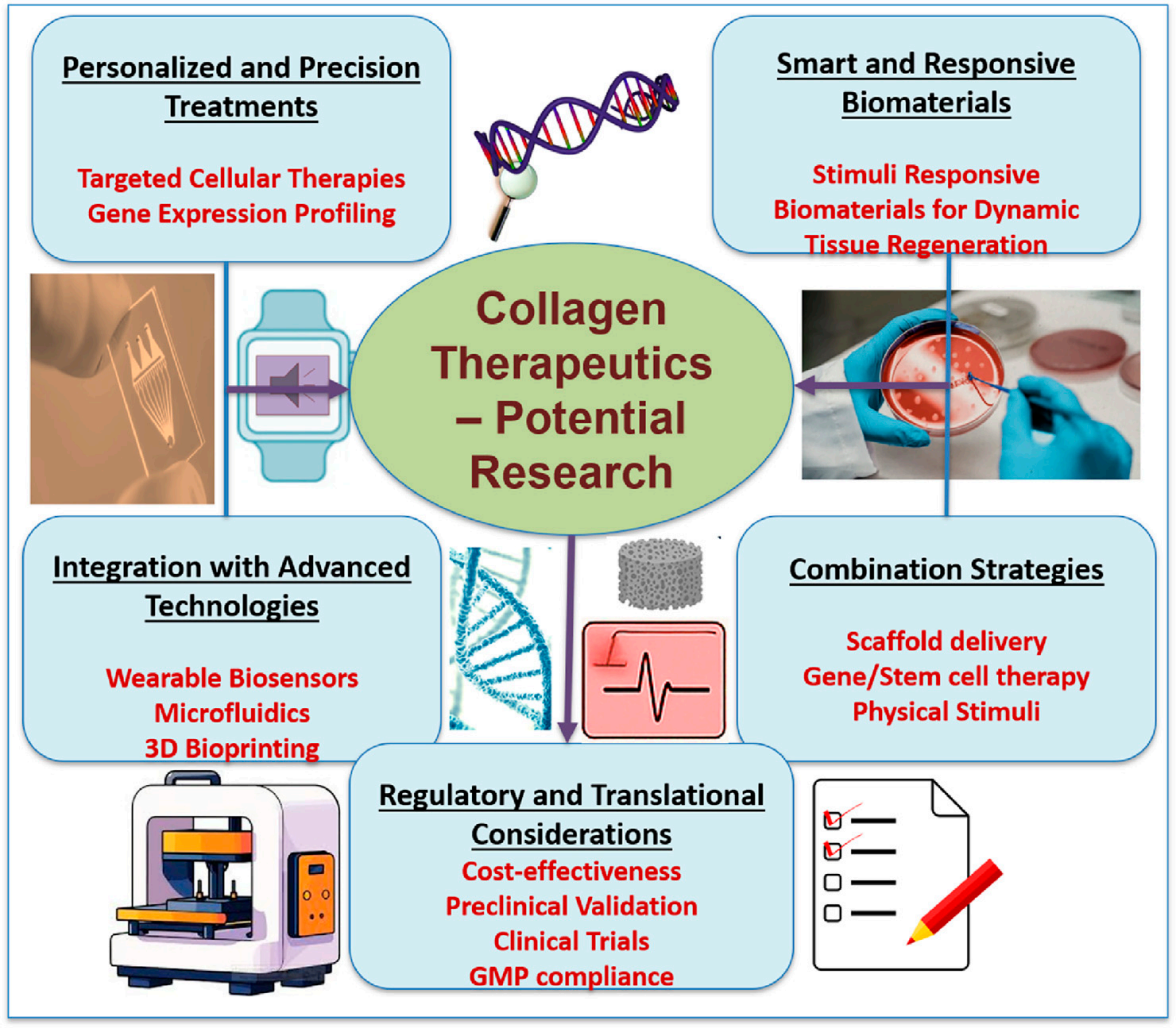
Future directions in collagen therapeutics, highlighting key areas in personalized treatments, smart biomaterials, integration of advanced technologies, combination strategies, and regulatory considerations. Emphasis is placed on targeted therapies, biosensor integration, scaffold-based delivery, and translational pathways for clinical application in regenerative medicine.

Collagen therapeutics is a rapidly emerging topic, propelled by interdisciplinary advancements in biomedical engineering, regenerative medicine, molecular biology, and material science. Several phenomena are anticipated to influence future developments in treatments for illnesses linked to collagen (Figure 11).

### 5.1 Personalized and precision medicine

The development of genomic and transcriptome profiling makes it possible to find collagen metabolism-related gene expression patterns and mutations unique to a patient. Gene editing, customized biomaterials, and customized cell therapies are examples of personalized therapeutic approaches explored to target specific illnesses more efficiently. Machine learning and bioinformatics developments also make it easier to forecast how a disease will advance and how therapy will work.

### 5.2 Smart and responsive biomaterials

The next-generation of biomaterials is designed to react to environmental stimuli like temperature, pH, enzymes, and mechanical stress (Siddiqua et al., 2024). These “smart” materials can direct tissue regeneration, adjust to changes in the wound microenvironment, and release bioactive chemicals dynamically. *In situ* modulation of collagen synthesis or incorporation into self-healing hydrogels, electroconductive scaffolds, and shape-memory polymers is a promising platform.

### 5.3 Integration with advanced technologies

The combination of collagen treatments with technologies like wearable sensors, microfluidics, and 3D bioprinting is opening new horizons in tissue engineering and diagnostics. Collagen-based bioinks in bioprinted tissues can replicate intricate topologies and facilitate *in vivo* functional integration (Marques et al., 2019). In disease models, microfluidic devices provide real-time collagen synthesis and breakdown monitoring. Wearable sensors may track ECM biomarkers noninvasively, allowing for dynamic treatment modifications.

### 5.4 Combination therapies

Combination therapies that combine scaffold-based delivery systems, gene and stem cell therapies, and physical stimulation techniques are anticipated in future clinical protocols. These integrative methods could synergistically aid tissue regeneration while overcome the drawbacks of single-modality treatments. For instance, a collagen-based hydrogel with microRNA therapies and stem cells, enhanced by localized electrical stimulation, may offer a multifaceted approach to healing chronic ulcers or regenerating pelvic floor tissue.

### 5.5 Regulatory and translational considerations

Regulatory frameworks must change to account for the complexity of bioengineered products to advance collagen-based medicines from the lab to the bedside. Robust preclinical validation, well-designed clinical trials, and adherence to good manufacturing practice (GMP) norms will be crucial (Granjeiro et al., 2024). To guarantee that these cutting-edge treatments are widely adopted, issues of cost-effectiveness, scalability, and patient accessibility must also be addressed.

## 6 Conclusion

Collagen plays a fundamental role in determining tissue structure, function, and regenerative capacity across multiple organ systems. Dysregulation of collagen homeostasis underlies a broad spectrum of pathological conditions, ranging from degenerative disorders such as osteoporosis and pelvic organ prolapse to malignancies including ovarian cancer. We emphasize the crucial role of collagen I/III ratio in ECM structural integrity and connective tissues’ biomechanics, and the diagnostic and therapeutic significance of the collagen I/III ratio for a wide range of pathological disorders. Understanding the molecular mechanisms governing collagen synthesis, degradation, and organization has catalyzed the development of innovative therapeutic strategies targeting these processes.

Recent advancements in biomaterials, stem cell biology, gene editing, and physical modulation are changing the therapeutic landscape for collagen disorders. These methods seek to restore native tissue functionality and mechanical integrity while replacing damaged or deficient collagen structures. As technological capabilities continue to evolve and our mechanistic understanding deepens, the clinical implementation of precision-engineered collagen therapeutics appears increasingly achievable. The utilization of collagen’s full therapeutic potential in regenerative medicine will require sustained interdisciplinary collaboration among researchers, clinicians, and regulatory specialists, coupled with robust translational research programs that effectively bridge laboratory discoveries and clinical applications. Through these coordinated efforts, we expect to optimize collagen-based interventions to significantly improve the quality of life for patients affected by the diverse spectrum of connective tissue disorders.

## References

[B1] Acri T. M. Laird N. Z. Jaidev L. R. Meyerholz D. K. Salem A. K. Shin K. (2021). Nonviral gene delivery embedded in biomimetically mineralized matrices for bone tissue engineering. Tissue Eng. Part A 27, 1074–1083. 10.1089/ten.tea.2020.0206 33086991 PMC8420947

[B2] Alkmin S. Patankar M. S. Campagnola P. J. (2022). Assessing the roles of collagen fiber morphology and matrix stiffness on ovarian cancer cell migration dynamics using multiphoton fabricated orthogonal image-based models. Acta Biomater. 153, 342–354. 10.1016/j.actbio.2022.09.037 36152908 PMC10324295

[B3] Amirrah I. N. Lokanathan Y. Zulkiflee I. Wee M. F. M. R. Motta A. Fauzi M. B. (2022). A comprehensive review on collagen type I development of biomaterials for tissue engineering: from biosynthesis to bioscaffold. Biomedicines 10, 2307. 10.3390/biomedicines10092307 36140407 PMC9496548

[B4] Anders J. J. Lanzafame R. J. Arany P. R. (2015). Low-level light/laser therapy versus photobiomodulation therapy. Photomed. Laser Surg. 33, 183–184. 10.1089/pho.2015.9848 25844681 PMC4390214

[B151] Antar S. A. Saleh M. A. Al-Karmalawy A. A. (2022). Investigating the possible mechanisms of pirfenidone to be targeted as a promising anti-inflammatory, anti-fibrotic, anti-oxidant, anti-apoptotic, anti-tumor, and/or anti-SARS-CoV-2. Life Sci. 309, 121048. 10.1016/j.lfs.2022.121048 36209833 PMC9536875

[B147] Aoki Y. (2024). Allele-specific CRISPR-Cas9 editing inactivates a single-nucleotide variant associated with collagen VI muscular dystrophy. Mol. Ther. Nucleic Acids 35. 10.1016/j.omtn.2024.102330 39380711 PMC11460449

[B5] Beam J. Botta A. Ye J. Soliman H. Matier B. J. Forrest M. (2015). Excess linoleic acid increases Collagen I/III ratio and “Stiffens” the heart muscle following high fat diets. J. Biol. Chem. 290, 23371–23384. 10.1074/jbc.M115.682195 26240151 PMC4645600

[B6] Bersie-Larson L. M. Gyoneva L. Goodman D. J. Dorfman K. D. Segal Y. Barocas V. H. (2020). Glomerular filtration and podocyte tensional homeostasis: importance of the minor type IV collagen network. Biomech. Model. Mechanobiol. 19, 2433–2442. 10.1007/s10237-020-01347-y 32462439 PMC7606712

[B7] Binlateh T. Thammanichanon P. Rittipakorn P. Thinsathid N. Jitprasertwong P. (2022). Collagen-based biomaterials in periodontal regeneration: current applications and future perspectives of plant-based collagen. Biomimetics 7, 34. 10.3390/biomimetics7020034 35466251 PMC9036199

[B8] Birch H. L. Thorpe C. T. Rumian A. P. (2013). Specialisation of extracellular matrix for function in tendons and ligaments. Muscles Ligaments Tendons J. 3, 12–22. 10.32098/mltj.01.2013.04 23885341 PMC3676159

[B9] Bretaud S. Guillon E. Karppinen S.-M. Pihlajaniemi T. Ruggiero F. (2020). Collagen XV, a multifaceted multiplexin present across tissues and species. Matrix Biol. 6-7 (6–7), 100023. 10.1016/j.mbplus.2020.100023 33543021 PMC7852327

[B10] Brown S. R. Cleveland E. M. Deeken C. R. Huitron S. S. Aluka K. J. Davis K. G. (2017). Type I/type III collagen ratio associated with diverticulitis of the colon in young patients. J. Surg. Res. 207, 229–234. 10.1016/j.jss.2016.08.044 27979482

[B11] Carroll L. O’Sullivan C. Doody C. Perrotta C. Fullen B. (2022). Pelvic organ prolapse: the lived experience. PLoS One 17, e0276788. 10.1371/journal.pone.0276788 36322592 PMC9629641

[B12] Cequera A. García de León Méndez M. C. (2014). Biomarkers for liver fibrosis: advances, advantages and disadvantages. Rev. Gastroenterol. Mex. 79, 187–199. 10.1016/j.rgmx.2014.05.003 24954541

[B13] Chi N. Wang R. (2018). Electrospun protein-CNT composite fibers and the application in fibroblast stimulation. Biochem. Biophys. Res. Commun. 504, 211–217. 10.1016/j.bbrc.2018.08.157 30172370

[B14] Chi N. Zheng S. Clutter E. Wang R. (2019). Silk-CNT mediated fibroblast stimulation toward chronic wound repair. Recent Prog. Mater. 1, 1. 10.21926/rpm.1904007 32550604 PMC7299232

[B15] Chi N. Lozo S. Rathnayake R. A. C. Botros-Brey S. Ma Y. Damaser M. (2022). Distinctive structure, composition and biomechanics of collagen fibrils in vaginal wall connective tissues associated with pelvic organ prolapse. Acta Biomater. 152, 335–344. 10.1016/j.actbio.2022.08.059 36055614 PMC10182770

[B16] Chuah Y. K. Basir R. Talib H. Tie T. H. Nordin N. (2013). Receptor for advanced glycation end products and its involvement in inflammatory diseases. Int. J. Inflamm. 2013, 1–15. 10.1155/2013/403460 24102034 PMC3786507

[B17] Chung S.-H. Kim W. B. (2018). Various approaches and treatments for pelvic organ prolapse in women. J. Menopausal Med. 24, 155–162. 10.6118/jmm.2018.24.3.155 30671407 PMC6336571

[B18] Chung H. J. Uitto J. (2010). Type VII collagen: the anchoring fibril protein at fault in dystrophic epidermolysis bullosa. Dermatol. Clin. 28, 93–105. 10.1016/j.det.2009.10.011 19945621 PMC2791403

[B19] Coolen A.-L. Lacroix C. Mercier-Gouy P. Delaune E. Monge C. Exposito J.-Y. (2019). Poly(lactic acid) nanoparticles and cell-penetrating peptide potentiate mRNA-based vaccine expression in dendritic cells triggering their activation. Biomaterials 195, 23–37. 10.1016/j.biomaterials.2018.12.019 30610991

[B20] Cross E. Prior J. A. Farmer A. D. Saunders B. (2023). Patients’ views and experiences of delayed diagnosis of inflammatory bowel disease: a qualitative study. BJGP Open 7. 10.3399/BJGPO.2023.0070 37549978 PMC11176705

[B21] Dasgupta I. McCollum D. (2019). Control of cellular responses to mechanical cues through YAP/TAZ regulation. J. Biol. Chem. 294, 17693–17706. 10.1074/jbc.REV119.007963 31594864 PMC6873206

[B22] Di Martino A. Cescon M. D’Agostino C. Schilardi F. Sabatelli P. Merlini L. (2023). Collagen VI in the musculoskeletal system. Int. J. Mol. Sci. 24, 5095. 10.3390/ijms24065095 36982167 PMC10049728

[B23] Eble J. A. Kassner A. Niland S. Mörgelin M. Grifka J. Grässel S. (2006). Collagen XVI harbors an integrin α1β1 recognition site in its C-terminal domains. J. Biol. Chem. 281, 25745–25756. 10.1074/jbc.M509942200 16754661

[B24] Erez N. Truitt M. Olson P. Arron S. T. Hanahan D. (2010). Cancer-associated fibroblasts are activated in incipient neoplasia to orchestrate tumor-promoting inflammation in an NF-κB-Dependent manner. Cancer Cell 17, 135–147. 10.1016/j.ccr.2009.12.041 20138012

[B25] Esquibel C. R. Wendt K. D. Lee H. C. Gaire J. Shoffstall A. Urdaneta M. E. (2020). Second harmonic generation imaging of collagen in chronically implantable electrodes in brain tissue. Front. Neurosci. 14, 95. 10.3389/fnins.2020.00095 32733179 PMC7358524

[B26] Ferreira J. P. S. Kuang M. Parente M. P. L. Natal Jorge R. M. Wang R. Eppell S. J. (2020). Altered mechanics of vaginal smooth muscle cells due to the lysyl oxidase-like1 knockout. Acta Biomater. 110, 175–187. 10.1016/j.actbio.2020.03.046 32335309

[B27] FitzGerald O. Gladman D. D. Mease P. J. Ritchlin C. Smolen J. S. Gao L. (2024). Phase 2 trial of deucravacitinib in psoriatic arthritis: biomarkers associated with disease activity, pharmacodynamics, and clinical responses. Arthritis Rheumatol. 76, 1397–1407. 10.1002/art.42921 38770592

[B28] Franco C. Patricia H.-R. Timo S. Claudia B. Marcela H. (2017). Matrix metalloproteinases as regulators of periodontal inflammation. Int. J. Mol. Sci. 18, 440. 10.3390/ijms18020440 28218665 PMC5343974

[B29] Gerrein A. Wright M. Cano-Sampaio N. Valle J. R. D. (2025). Synthesis and stability of collagen mimetic peptides featuring δ-heteroatom-substituted prolines. Org. Biomol. Chem. 23, 3097–3101. 10.1039/D5OB00176E 40019362

[B30] Gillesberg F. S. Lindholm M. Karsdal M. A. Bay-Jensen A. C. Manon-Jensen T. Sun S. (2024). “Chapter 14 - type XIV collagen,” in Biochemistry of collagens, laminins and elastin. Third Edition (Academic Press), 131–135. 10.1016/B978-0-443-15617-5.00049-4

[B31] Giubertoni G. Feng L. Klein K. Giannetti G. Rutten L. Choi Y. (2024). Elucidating the role of water in collagen self-assembly by isotopically modulating collagen hydration. Proc. Natl. Acad. Sci. 121, e2313162121. 10.1073/pnas.2313162121 38451946 PMC10945838

[B32] Granjeiro J. M. Borchio P. G. de M. Ribeiro I. P. B. Paiva K. B. S. (2024). Bioengineering breakthroughs: the impact of stem cell models on advanced therapy medicinal product development. World J. Stem Cells 16, 860–872. 10.4252/wjsc.v16.i10.860 39493828 PMC11525646

[B33] Gretzmeier C. Pin D. Kern J. S. Chen M. Woodley D. T. Bruckner-Tuderman L. (2022). Systemic collagen VII replacement therapy for advanced recessive dystrophic epidermolysis bullosa. J. Invest. Dermatol. 142, 1094–1102.e3. 10.1016/j.jid.2021.09.008 34606885

[B34] Guillon C. Ferraro S. Clément S. Bouschbacher M. Sigaudo-Roussel D. Bonod C. (2021). Glycation by glyoxal leads to profound changes in the behavior of dermal fibroblasts. BMJ Open Diabetes Res. 9, e002091. 10.1136/bmjdrc-2020-002091 33903117 PMC8076933

[B35] Hamblin M. R. (2017). Mechanisms and applications of the anti-inflammatory effects of photobiomodulation. AIMS Biophys. 4, 337–361. 10.3934/biophy.2017.3.337 28748217 PMC5523874

[B36] Heikkinen A. Härönen H. Norman O. Pihlajaniemi T. (2020). Collagen XIII and other ECM components in the assembly and disease of the neuromuscular junction. Anat. Rec. 303, 1653–1663. 10.1002/ar.24092 30768864

[B37] Heilig J. Dietmar H. F. Brachvogel B. Paulsson M. Zaucke F. Niehoff A. (2020). Collagen IX deficiency leads to premature vascularization and ossification of murine femoral heads through an imbalance of pro- and antiangiogenic factors. Osteoarthr. Cartil. 28, 988–999. 10.1016/j.joca.2020.03.015 32283184

[B38] Henriksen K. Karsdal M. A. (2024). “Chapter 1 - type I collagen,” in Biochemistry of collagens, laminins and elastin. Third Edition (Academic Press), 1–11. 10.1016/B978-0-443-15617-5.00047-0

[B39] Herberts C. A. Kwa M. S. Hermsen H. P. (2011). Risk factors in the development of stem cell therapy. J. Transl. Med. 9, 29. 10.1186/1479-5876-9-29 21418664 PMC3070641

[B152] Herbertz S. Sawyer J. S. Stauber A. J. Gueorguieva I. Driscoll K. E. Estrem S. T. (2015). Clinical development of galunisertib (LY2157299 monohydrate), a small molecule inhibitor of transforming growth factor-beta signaling pathway. Drug Des. Devel. Ther. 9, 4479–4499. 10.2147/DDDT.S86621 26309397 PMC4539082

[B40] Herchenhan A. Uhlenbrock F. Eliasson P. Weis M. Eyre D. Kadler K. E. (2015). Lysyl oxidase activity is required for ordered collagen fibrillogenesis by tendon cells. J. Biol. Chem. 290, 16440–16450. 10.1074/jbc.M115.641670 25979340 PMC4481240

[B41] Holl J. Kowalewski C. Zimek Z. Fiedor P. Kaminski A. Oldak T. (2021). Chronic diabetic wounds and their treatment with skin substitutes. Cells 10, 655. 10.3390/cells10030655 33804192 PMC8001234

[B42] Huang S. Li Q. Li X. Ye H. Zhang L. Zhu X. (2025). Recent research progress of wound healing biomaterials containing platelet-rich plasma. Int. J. Nanomedicine 20, 3961–3976. 10.2147/IJN.S506677 40191044 PMC11970316

[B43] Huebner P. Warren P. B. Chester D. Spang J. T. Brown A. C. Fisher M. B. (2020). Mechanical properties of tissue formed *in vivo* are affected by 3D-bioplotted scaffold microarchitecture and correlate with ECM collagen fiber alignment. Connect. Tissue Res. 61, 190–204. 10.1080/03008207.2019.1624733 31345062

[B44] Isler S. C. Soysal F. Ceyhanlı T. Bakırarar B. Unsal B. (2022). Efficacy of concentrated growth factor *versus* collagen membrane in reconstructive surgical therapy of peri-implantitis: 3-year results of a randomized clinical trial. Clin. Oral Investig. 26, 5247–5260. 10.1007/s00784-022-04493-y 35618961 PMC9381616

[B45] Izu Y. Adams S. M. Connizzo B. K. Beason D. P. Soslowsky L. J. Koch M. (2021). Collagen XII mediated cellular and extracellular mechanisms regulate establishment of tendon structure and function. Matrix Biol. J. Int. Soc. Matrix Biol. 95, 52–67. 10.1016/j.matbio.2020.10.004 33096204 PMC7870578

[B46] Jameson S. A. Swaminathan G. Dahal S. Couri B. Kuang M. Rietsch A. (2020). Elastin homeostasis is altered with pelvic organ prolapse in cultures of vaginal cells from a lysyl oxidase‐like 1 knockout mouse model. Physiol. Rep. 8, e14436. 10.14814/phy2.14436 32533648 PMC7292929

[B47] Jiang Y. Zhang H. Wang J. Liu Y. Luo T. Hua H. (2022). Targeting extracellular matrix stiffness and mechanotransducers to improve cancer therapy. J. Hematol. Oncol. 15, 34. 10.1186/s13045-022-01252-0 35331296 PMC8943941

[B48] Jonidi Shariatzadeh F. Currie S. Logsetty S. Spiwak R. Liu S. (2025). Enhancing wound healing and minimizing scarring: a comprehensive review of nanofiber technology in wound dressings. Prog. Mater. Sci. 147, 101350. 10.1016/j.pmatsci.2024.101350

[B148] Jung H. Rim Y. A. Park N. Nam Y. Ju J. H. (2021). Restoration of osteogenesis by CRISPR/Cas9 genome editing of the mutated COL1A1 gene in psteogenesis imperfecta. J. Clin. Med. 10, 3141. 10.3390/jcm10143141 34300306 PMC8307903

[B49] Jürgensen H. J. van Putten S. Nørregaard K. S. Bugge T. H. Engelholm L. H. Behrendt N. (2020). Cellular uptake of collagens and implications for immune cell regulation in disease. Cell. Mol. Life Sci. CMLS 77, 3161–3176. 10.1007/s00018-020-03481-3 32100084 PMC11105017

[B50] Kaku M. Yamauchi M. (2014). Mechano-regulation of collagen biosynthesis in periodontal ligament. J. Prosthodont. Res. 58, 193–207. 10.1016/j.jpor.2014.08.003 25311991 PMC4253671

[B51] Kamakura T. Jin Y. Nishio M. Nagata S. Fukuda M. Sun L. (2023). Collagen X is dispensable for hypertrophic differentiation and endochondral ossification of human iPSC‐derived chondrocytes. JBMR Plus 7, e10737. 10.1002/jbm4.10737 37197316 PMC10184020

[B52] Kanniyappan H. Thangavel P. Chakraborty S. Arige V. Muthuvijayan V. (2020). Design and evaluation of Konjac glucomannan-based bioactive interpenetrating network (IPN) scaffolds for engineering vascularized bone tissues. Int. J. Biol. Macromol. 143, 30–40. 10.1016/j.ijbiomac.2019.12.012 31811851

[B53] Kanniyappan H. Venkatesan M. Panji J. Ramasamy M. Muthuvijayan V. (2021). Evaluating the inherent osteogenic and angiogenic potential of mesoporous silica nanoparticles to augment vascularized bone tissue formation. Microporous Mesoporous Mater 311, 110687. 10.1016/j.micromeso.2020.110687

[B54] Kanniyappan H. Gnanasekar V. Parise V. Debnath K. Sun Y. Thakur S. (2024a). Harnessing extracellular vesicles-mediated signaling for enhanced bone regeneration: novel insights into scaffold design. Biomed. Mater. Bristol Engl. 19, 055004. 10.1088/1748-605X/ad5ba9 38917828 PMC11305091

[B55] Kanniyappan H. Sundaram M. K. Ravikumar A. Chakraborty S. Gnanamani A. Mani U. (2024b). Enhancing bone repair through improved angiogenesis and osteogenesis using mesoporous silica nanoparticle-loaded Konjac glucomannan-based interpenetrating network scaffolds. Int. J. Biol. Macromol. 279, 135182. 10.1016/j.ijbiomac.2024.135182 39216566

[B56] Karamanos N. K. Theocharis A. D. Piperigkou Z. Manou D. Passi A. Skandalis S. S. (2021). A guide to the composition and functions of the extracellular matrix. FEBS J. 288, 6850–6912. 10.1111/febs.15776 33605520

[B57] Karsdal M. (2023). Biochemistry of collagens, laminins and elastin: structure, function and biomarkers. Elsevier.

[B58] Kelly E. C. Winick-Ng J. Welk B. (2016). Surgeon experience and complications of transvaginal prolapse mesh. Obstet. Gynecol. 128, 65–72. 10.1097/AOG.0000000000001450 27275803

[B59] Kim T. Sridharan I. Zhu B. Orgel J. Wang R. (2015). Effect of CNT on collagen fiber structure, stiffness assembly kinetics and stem cell differentiation. Mater. Sci. Eng. C 49, 281–289. 10.1016/j.msec.2015.01.014 25686951 PMC7225775

[B60] Kim T. Sridharan I. Ma Y. Zhu B. Chi N. Kobak W. (2016). Identifying distinct nanoscopic features of native collagen fibrils towards early diagnosis of pelvic organ prolapse. Nanomedicine Nanotechnol. Biol. Med. 12, 667–675. 10.1016/j.nano.2015.11.006 26656625

[B149] Kole R. Krainer A. R. Altman S. (2012). RNA therapeutics: Beyond RNA interference and antisense oligonucleotides. Nat. Rev. Drug Discov. 11, 125–140. 10.1038/nrd3625 22262036 PMC4743652

[B61] Kuivaniemi H. Tromp G. (2019). Type III collagen (COL3A1): Gene and protein structure, tissue distribution, and associated diseases. Gene 707, 151–171. 10.1016/j.gene.2019.05.003 31075413 PMC6579750

[B62] Kwon T. Lamster I. B. Levin L. (2021). Current concepts in the management of periodontitis. Int. Dent. J. 71, 462–476. 10.1111/idj.12630 34839889 PMC9275292

[B63] Lai C.-S. Tu C.-W. Kuo H.-C. Sun P.-P. Tsai M.-L. (2020). Type II collagen from cartilage of *Acipenser baerii* promotes wound healing in human dermal fibroblasts and in mouse skin. Mar. Drugs 18, 511. 10.3390/md18100511 33050593 PMC7601416

[B64] Levental K. R. Yu H. Kass L. Lakins J. N. Egeblad M. Erler J. T. (2009). Matrix crosslinking forces tumor progression by enhancing integrin signaling. Cell 139, 891–906. 10.1016/j.cell.2009.10.027 19931152 PMC2788004

[B65] Li W. Zhu B. Strakova Z. Wang R. (2014). Two-way regulation between cells and aligned collagen fibrils: local 3D matrix formation and accelerated neural differentiation of human decidua parietalis placental stem cells. Biochem. Biophys. Res. Commun. 450, 1377–1382. 10.1016/j.bbrc.2014.06.136 25003322 PMC4134991

[B66] Li W. Liu Y. Zhang P. Tang Y. Zhou M. Jiang W. (2018). Tissue-engineered bone immobilized with human adipose stem cells-derived exosomes promotes bone regeneration. ACS Appl. Mater. Interfaces 10, 5240–5254. 10.1021/acsami.7b17620 29359912

[B67] Li Z. Yu M. Jin S. Wang Y. Luo R. Huo B. (2019). Stress distribution and collagen remodeling of periodontal ligament during orthodontic tooth movement. Front. Pharmacol. 10, 1263. 10.3389/fphar.2019.01263 31708784 PMC6821875

[B68] Li W. Chi N. Clutter E. D. Zhu B. Wang R. R. (2021a). Aligned Collagen-CNT nanofibrils and the modulation effect on ovarian cancer cells. J. Compos. Sci. 5, 148. 10.3390/jcs5060148 35664989 PMC9164112

[B69] Li W. Chi N. Rathnayake R. A. C. Wang R. (2021b). Distinctive roles of fibrillar collagen I and collagen III in mediating fibroblast-matrix interaction: a nanoscopic study. Biochem. Biophys. Res. Commun. 560, 66–71. 10.1016/j.bbrc.2021.04.088 33975247 PMC8165026

[B70] Li S. Li X. Xu Y. Fan C. Li Z. A. Zheng L. (2024). Collagen fibril-like injectable hydrogels from self-assembled nanoparticles for promoting wound healing. Bioact. Mater. 32, 149–163. 10.1016/j.bioactmat.2023.09.012 37822915 PMC10563012

[B71] Liang Y. Lv Z. Huang G. Qin J. Li H. Nong F. (2020). Prognostic significance of abnormal matrix collagen remodeling in colorectal cancer based on histologic and bioinformatics analysis. Oncol. Rep. 44, 1671–1685. 10.3892/or.2020.7729 32945508 PMC7448414

[B72] Liu J. Khalil R. A. (2017). Matrix metalloproteinase inhibitors as investigational and therapeutic tools in unrestrained tissue remodeling and pathological disorders. Prog. Mol. Biol. Transl. Sci. 148, 355–420. 10.1016/bs.pmbts.2017.04.003 28662828 PMC5548434

[B73] Lu Y. Zhang S. Wang Y. Ren X. Han J. (2019). Molecular mechanisms and clinical manifestations of rare genetic disorders associated with type I collagen. Intractable Rare Dis. Res. 8, 98–107. 10.5582/irdr.2019.01064 31218159 PMC6557237

[B74] Luchian I. Goriuc A. Sandu D. Covasa M. (2022). The role of matrix metalloproteinases (MMP-8, MMP-9, MMP-13) in periodontal and peri-implant pathological processes. Int. J. Mol. Sci. 23, 1806. 10.3390/ijms23031806 35163727 PMC8837018

[B75] Mäkitie R. E. Costantini A. Kämpe A. Alm J. J. Mäkitie O. (2019). New insights into monogenic causes of osteoporosis. Front. Endocrinol. 10, 70. 10.3389/fendo.2019.00070 30858824 PMC6397842

[B76] Marques C. F. Diogo G. S. Pina S. Oliveira J. M. Silva T. H. Reis R. L. (2019). Collagen-based bioinks for hard tissue engineering applications: a comprehensive review. J. Mater. Sci. Mater. Med. 30, 32. 10.1007/s10856-019-6234-x 30840132

[B77] Mgwenya T. N. Abrahamse H. Houreld N. N. (2025). Photobiomodulation studies on diabetic wound healing: an insight into the inflammatory pathway in diabetic wound healing. Wound Repair Regen. 33, e13239. 10.1111/wrr.13239 39610015 PMC11628774

[B78] Mienaltowski M. J. Gonzales N. L. Beall J. M. Pechanec M. Y. (2021). “Basic structure, physiology, and biochemistry of connective tissues and extracellular matrix collagens,” in Progress in heritable soft connective tissue diseases. Editor Halper J. (Cham: Springer International Publishing), 5–43. 10.1007/978-3-030-80614-9_2 34807414

[B79] Mohabeer A. L. Kroetsch J. T. McFadden M. Khosraviani N. Broekelmann T. J. Hou G. (2021). Deletion of type VIII collagen reduces blood pressure, increases carotid artery functional distensibility and promotes elastin deposition. Matrix Biol. Plus 12, 100085. 10.1016/j.mbplus.2021.100085 34693248 PMC8517381

[B80] Mukherjee A. Das B. (2024). The role of inflammatory mediators and matrix metalloproteinases (MMPs) in the progression of osteoarthritis. Biomater. Biosyst. 13, 100090. 10.1016/j.bbiosy.2024.100090 38440290 PMC10910010

[B81] Munezane H. Oizumi H. Wakabayashi T. Nishio S. Hirasawa T. Sato T. (2019). Roles of collagen XXV and its putative receptors PTPσ/δ in intramuscular motor innervation and congenital cranial dysinnervation disorder. Cell Rep. 29, 4362–4376.e6. 10.1016/j.celrep.2019.11.112 31875546

[B82] Muona A. Eklund L. Väisänen T. Pihlajaniemi T. (2002). Developmentally regulated expression of type XV collagen correlates with abnormalities in Col15a1(-/-) mice. Matrix Biol. J. Int. Soc. Matrix Biol. 21, 89–102. 10.1016/s0945-053x(01)00187-1 11827796

[B83] Nam J.-S. Terabe M. Mamura M. Kang M.-J. Chae H. Stuelten C. (2008). An anti–transforming growth factor β antibody suppresses metastasis *via* cooperative effects on multiple cell compartments. Cancer Res. 68, 3835–3843. 10.1158/0008-5472.CAN-08-0215 18483268 PMC2587151

[B84] Narauskaitė D. Vydmantaitė G. Rusteikaitė J. Sampath R. Rudaitytė A. Stašytė G. (2021). Extracellular vesicles in skin wound healing. Pharmaceuticals 14, 811. 10.3390/ph14080811 34451909 PMC8400229

[B85] Natarajan S. Foreman K. M. Soriano M. I. Rossen N. S. Shehade H. Fregoso D. R. (2019). Collagen remodeling in the hypoxic tumor-mesothelial niche promotes ovarian cancer metastasis. Cancer Res. 79, 2271–2284. 10.1158/0008-5472.CAN-18-2616 30862717 PMC6822898

[B86] Orgel J. P. R. O. Antipova O. Sagi I. Bitler A. Qiu D. Wang R. (2011). Collagen fibril surface displays a constellation of sites capable of promoting fibril assembly, stability, and hemostasis. Connect. Tissue Res. 52, 18–24. 10.3109/03008207.2010.511354 21117898 PMC3244825

[B87] Oyebode O. A. Jere S. W. Houreld N. N. (2023). Current therapeutic modalities for the management of chronic diabetic wounds of the foot. J. Diabetes Res. 2023, 1–10. 10.1155/2023/1359537 36818748 PMC9937766

[B88] O’Connell K. S. (2014). Investigation of selected collagen genes in exercise-related musculoskeletal soft tissue phenotypes.

[B89] Panwar P. Butler G. S. Jamroz A. Azizi P. Overall C. M. Brömme D. (2018). Aging-associated modifications of collagen affect its degradation by matrix metalloproteinases. Matrix Biol. 65, 30–44. 10.1016/j.matbio.2017.06.004 28634008

[B90] Park H. J. Rouabhia M. Lavertu D. Zhang Z. (2015). Electrical stimulation modulates the expression of multiple wound healing genes in primary human dermal fibroblasts. Tissue Eng. Part A 21, 1982–1990. 10.1089/ten.TEA.2014.0687 25873313

[B91] Peng Q. Qian Y. Xiao X. Gao F. Ren G. Pennisi C. P. (2025). Advancing chronic wound healing through electrical stimulation and adipose‐derived stem cells. Adv. Healthc. Mater. 14, 2403777. 10.1002/adhm.202403777 40025921 PMC12004429

[B92] Persu C. Chapple C. Cauni V. Gutue S. Geavlete P. (2011). Pelvic organ prolapse quantification system (POP–Q) – a new era in pelvic prolapse staging. J. Med. Life 4, 75–81. Available online at: https://pmc.ncbi.nlm.nih.gov/articles/PMC3056425/ . 21505577 PMC3056425

[B93] Perumal S. Antipova O. Orgel J. P. R. O. (2008). Collagen fibril architecture, domain organization, and triple-helical conformation govern its proteolysis. Proc. Natl. Acad. Sci. 105, 2824–2829. 10.1073/pnas.0710588105 18287018 PMC2268544

[B94] Pickup M. W. Laklai H. Acerbi I. Owens P. Gorska A. E. Chytil A. (2013). Stromally derived lysyl oxidase promotes metastasis of transforming growth factor-β-deficient mouse mammary carcinomas. Cancer Res. 73, 5336–5346. 10.1158/0008-5472.CAN-13-0012 23856251 PMC3766496

[B95] Pires J. A. Bragato E. F. Momolli M. Guerra M. B. Neves L. M. Bruscagnin M. A. de O. (2022). Effect of the combination of photobiomodulation therapy and the intralesional administration of corticoid in the preoperative and postoperative periods of keloid surgery: a randomized, controlled, double-blind trial protocol study. PLOS ONE 17, e0263453. 10.1371/journal.pone.0263453 35167583 PMC8846523

[B96] Quan J. Yahata T. Adachi S. Yoshihara K. Tanaka K. (2011). Identification of receptor tyrosine kinase, discoidin domain receptor 1 (DDR1), as a potential biomarker for serous ovarian cancer. Int. J. Mol. Sci. 12, 971–982. 10.3390/ijms12020971 21541037 PMC3083684

[B97] Rathnayake R. A. C. Yoon S. Zheng S. Clutter E. D. Wang R. R. (2022). Electrospun silk Fibroin-CNT composite fibers: characterization and application in mediating fibroblast stimulation. Polymers 15, 91. 10.3390/polym15010091 36616441 PMC9824115

[B98] Rinaldi F. Pinto D. Trink A. Giuliani G. Sparavigna A. (2021). *In vitro* and *in vivo* evaluation on the safety and efficacy of a Brand-new intracutaneous filler with α1-R-Collagen. Clin. Cosmet. Investig. Dermatol. 14, 501–512. 10.2147/CCID.S295618 34012283 PMC8126805

[B99] Seppinen L. Sormunen R. Soini Y. Elamaa H. Heljasvaara R. Pihlajaniemi T. (2008). Lack of collagen XVIII accelerates cutaneous wound healing, while overexpression of its endostatin domain leads to delayed healing. Matrix Biol. 27, 535–546. 10.1016/j.matbio.2008.03.003 18455382

[B100] Shabani Z. Schuerger J. Zhu X. Tang C. Ma L. Yadav A. (2024). Increased collagen I/Collagen III ratio is associated with hemorrhage in brain arteriovenous malformations in human and mouse. Cells 13, 92. 10.3390/cells13010092 38201296 PMC10778117

[B101] Shen G. (2005). The role of type X collagen in facilitating and regulating endochondral ossification of articular cartilage. Orthod. Craniofac. Res. 8, 11–17. 10.1111/j.1601-6343.2004.00308.x 15667640

[B102] Siddiqua A. Clutter E. Garklavs O. Kanniyappan H. Wang R. R. (2024). Electrospun Silk-ICG composite fibers and the application toward hemorrhage control. J. Funct. Biomater. 15, 272. 10.3390/jfb15090272 39330247 PMC11433354

[B103] Silver F. H. Kelkar N. Deshmukh T. (2021). Molecular basis for mechanical properties of ECMs: proposed role of fibrillar collagen and Proteoglycans in tissue biomechanics. Biomolecules 11, 1018. 10.3390/biom11071018 34356642 PMC8301845

[B104] Singh D. Rai V. Agrawal D. K. (2023). Regulation of collagen I and collagen III in tissue injury and regeneration. Cardiol. Cardiovasc. Med. 7, 5–16. 10.26502/fccm.92920302 36776717 PMC9912297

[B105] Sridharan I. Kim T. Wang R. (2009). Adapting collagen/CNT matrix in directing hESC differentiation. Biochem. Biophys. Res. Commun. 381, 508–512. 10.1016/j.bbrc.2009.02.072 19233124 PMC2703609

[B106] Sridharan I. Ma Y. Kim T. Kobak W. Rotmensch J. Wang R. (2012). Structural and mechanical profiles of native collagen fibers in vaginal wall connective tissues. Biomaterials 33, 1520–1527. 10.1016/j.biomaterials.2011.11.005 22112762

[B107] Sridharan I. Kim T. Strakova Z. Wang R. (2013). Matrix-specified differentiation of human decidua parietalis placental stem cells. Biochem. Biophys. Res. Commun. 437, 489–495. 10.1016/j.bbrc.2013.07.002 23850689 PMC3913263

[B108] Staneva R. Burla F. Koenderink G. H. Descroix S. Vignjevic D. M. Attieh Y. (2018). A new biomimetic assay reveals the temporal role of matrix stiffening in cancer cell invasion. Mol. Biol. Cell 29, 2979–2988. 10.1091/mbc.E18-01-0068 30303750 PMC6333180

[B109] Subramanian S. Anastasopoulou C. Viswanathan V. K. (2025). “Osteogenesis imperfecta,” in *StatPearls*, treasure Island, FL (StatPearls Publishing). Available online at: http://www.ncbi.nlm.nih.gov/books/NBK536957/ (Accessed May 22, 2025). 30725642

[B110] Südy R. Peták F. Kiss L. Balogh Á. L. Fodor G. H. Korsós A. (2021). Obesity and diabetes: similar respiratory mechanical but different gas exchange defects. Am. J. Physiol. Lung Cell. Mol. Physiol. 320, L368–L376. 10.1152/ajplung.00439.2020 33264577

[B111] Sun M. Luo E. Y. Adams S. M. Adams T. Ye Y. Shetye S. S. (2020). Collagen XI regulates the acquisition of collagen fibril structure, organization and functional properties in tendon. Matrix Biol. J. Int. Soc. Matrix Biol. 94, 77–94. 10.1016/j.matbio.2020.09.001 32950601 PMC7722227

[B112] Sun Y. Li L. Wang J. Liu H. Wang H. (2024). Emerging landscape of osteogenesis imperfecta pathogenesis and therapeutic approaches. ACS Pharmacol. Transl. Sci. 7, 72–96. 10.1021/acsptsci.3c00324 38230285 PMC10789133

[B113] Surdiacourt L. Christiaens V. De Bruyckere T. De Buyser S. Eghbali A. Vervaeke S. (2025). A multi-centre randomized controlled trial comparing connective tissue graft with collagen matrix to increase soft tissue thickness at the buccal aspect of single implants: 3-year results. J. Clin. Periodontol. 52, 92–101. 10.1111/jcpe.13975 38485651

[B114] Takallu S. Kakian F. Bazargani A. Khorshidi H. Mirzaei E. (2024). Development of antibacterial collagen membranes with optimal silver nanoparticle content for periodontal regeneration. Sci. Rep. 14, 7262. 10.1038/s41598-024-57951-w 38538709 PMC10973344

[B115] Thorseth M.-L. Carretta M. Jensen C. Mølgaard K. Jürgensen H. J. Engelholm L. H. (2022). Uncovering mediators of collagen degradation in the tumor microenvironment. Matrix Biol. Plus 13, 100101. 10.1016/j.mbplus.2022.100101 35198964 PMC8841889

[B116] Tresoldi I. Oliva F. Benvenuto M. Fantini M. Masuelli L. Bei R. (2013). Tendon’s ultrastructure. Muscles Ligaments Tendons J. 3, 2–6. 10.32098/mltj.01.2013.02 23885339 PMC3676160

[B117] Uitterlinden A. G. Burger H. Huang Q. Yue F. McGuigan F. E. A. Grant S. F. A. (1998). Relation of alleles of the collagen type Iα1 gene to bone density and the risk of osteoporotic fractures in postmenopausal women. N. Engl. J. Med. 338, 1016–1021. 10.1056/NEJM199804093381502 9535665

[B118] van Leusden M. R. Pas H. H. Gedde-Dahl T. Sonnenberg A. Jonkman M. F. (2001). Truncated type XVII collagen expression in a patient with non-herlitz junctional epidermolysis bullosa caused by a homozygous splice-site mutation. Lab. Invest. 81, 887–894. 10.1038/labinvest.3780297 11406649

[B150] Wang B. Komers R. Carew R. Winbanks C. E. Xu B. Herman-Edelstein M. (2012). Suppression of microRNA-29 expression by TGF-β1 promotes collagen expression and renal fibrosis. J. Am. Soc. Nephrol. 23, 252–265. 10.1681/ASN.2011010055 22095944 PMC3269175

[B119] Wang T. Zhou Z. Luo E. Zhong J. Zhao D. Dong H. (2021). Comprehensive RNA sequencing in primary murine keratinocytes and fibroblasts identifies novel biomarkers and provides potential therapeutic targets for skin-related diseases. Cell. Mol. Biol. Lett. 26, 42. 10.1186/s11658-021-00285-6 34602061 PMC8489068

[B120] Wang B. Chen Y. Zhu X. Wang T. Li M. Huang Y. (2022a). Global burden and trends of pelvic organ prolapse associated with aging women: an observational trend study from 1990 to 2019. Front. Public Health 10, 975829. 10.3389/fpubh.2022.975829 36187690 PMC9521163

[B121] Wang J. Hu H. Wang J. Qiu H. Gao Y. Xu Y. (2022b). Characterization of recombinant humanized collagen type III and its influence on cell behavior and phenotype. J. Leather Sci. Eng. 4, 33. 10.1186/s42825-022-00103-5

[B122] Wang S. Fu Y. Kuerban K. Liu J. Huang X. Pan D. (2022c). Discoidin domain receptor 1 is a potential target correlated with tumor invasion and immune infiltration in gastric cancer. Front. Immunol. 13, 933165. 10.3389/fimmu.2022.933165 35935941 PMC9353406

[B123] Wang X. He R. Nian S. Xiao B. Wang Y. Zhang L. (2022d). Treatment of pelvic organ prolapse by the downregulation of the expression of mitofusin 2 in uterosacral ligament tissue *via* mesenchymal stem cells. Genes 13, 829. 10.3390/genes13050829 35627214 PMC9141332

[B124] Wen C.-Y. Wu C.-B. Tang B. Wang T. Yan C.-H. Lu W. W. (2012). Collagen fibril stiffening in osteoarthritic cartilage of human beings revealed by atomic force microscopy. Osteoarthr. Cartil. 20, 916–922. 10.1016/j.joca.2012.04.018 22548795

[B125] Wen S. Zheng X. Yin W. Liu Y. Wang R. Zhao Y. (2024). Dental stem cell dynamics in periodontal ligament regeneration: from mechanism to application. Stem Cell Res. Ther. 15, 389. 10.1186/s13287-024-04003-9 39482701 PMC11526537

[B126] Wenstrup R. J. Smith S. M. Florer J. B. Zhang G. Beason D. P. Seegmiller R. E. (2011). Regulation of collagen fibril nucleation and initial fibril assembly involves coordinate interactions with collagens V and XI in developing tendon. J. Biol. Chem. 286, 20455–20465. 10.1074/jbc.M111.223693 21467034 PMC3121453

[B127] Winer A. Adams S. Mignatti P. (2018). Matrix metalloproteinase inhibitors in cancer therapy: turning past failures into future successes. Mol. Cancer Ther. 17, 1147–1155. 10.1158/1535-7163.MCT-17-0646 29735645 PMC5984693

[B128] Wittig C. Szulcek R. (2021). Extracellular matrix protein ratios in the human heart and vessels: how to distinguish pathological from physiological changes? Front. Physiol. 12, 708656. 10.3389/fphys.2021.708656 34421650 PMC8371527

[B129] Woo K. (2024). Photobiomodulation as a multimodal therapy to enhance wound healing and skin regeneration. Med. Lasers Eng. Basic Res. Clin. Appl. 13, 173–184. 10.25289/ML.24.033

[B130] Wu J. M. Dieter A. A. Pate V. Jonsson Funk M. (2017). Cumulative incidence of a subsequent surgery after stress urinary incontinence and pelvic organ prolapse procedure. Obstet. Gynecol. 129, 1124–1130. 10.1097/AOG.0000000000002051 28486368 PMC5774231

[B131] Wu D. Ding Z. Lu T. Chen Y. Zhang F. Lu S. (2024). DDR1-targeted therapies: current limitations and future potential. Drug Discov. Today 29, 103975. 10.1016/j.drudis.2024.103975 38580164

[B132] Xu Q. Torres J. E. Hakim M. Babiak P. M. Pal P. Battistoni C. M. (2021). Collagen- and hyaluronic acid-based hydrogels and their biomedical applications. Mater. Sci. Eng. R. Rep. 146, 100641. 10.1016/j.mser.2021.100641 34483486 PMC8409465

[B133] Xu J. Jia Y. Huang W. Shi Q. Sun X. Zheng L. (2022). Non-contact electrical stimulation as an effective means to promote wound healing. Bioelectrochemistry 146, 108108. 10.1016/j.bioelechem.2022.108108 35366594

[B134] Xu L.-M. Yu X.-X. Zhang N. Chen Y.-S. (2024). Exosomes from umbilical cord mesenchymal stromal cells promote the collagen production of fibroblasts from pelvic organ prolapse. World J. Stem Cells 16, 708–727. 10.4252/wjsc.v16.i6.708 38948096 PMC11212552

[B135] Yamauchi M. Sricholpech M. (2012). Lysine post-translational modifications of collagen. Essays Biochem. 52, 113–133. 10.1042/bse0520113 22708567 PMC3499978

[B136] Yang Y. Yang H. H. Tang B. Lai Wu A. M. Flanders K. C. Moshkovich N. (2020). The outcome of TGFβ antagonism in metastatic breast cancer models *in vivo* reflects a complex balance between tumor-suppressive and pro-progression activities of TGFβ. Clin. Cancer Res. Off. J. Am. Assoc. Cancer Res. 26, 643–656. 10.1158/1078-0432.CCR-19-2370 31582516 PMC8182485

[B137] Yellon S. M. (2020). Immunobiology of cervix ripening. Front. Immunol. 10, 3156. 10.3389/fimmu.2019.03156 32038651 PMC6993120

[B138] Yen Y.-H. Pu C.-M. Liu C.-W. Chen Y.-C. Chen Y.-C. Liang C.-J. (2018). Curcumin accelerates cutaneous wound healing *via* multiple biological actions: the involvement of TNF-α, MMP-9, α-SMA, and collagen. Int. Wound J. 15, 605–617. 10.1111/iwj.12904 29659146 PMC7950016

[B139] Young B. B. Gordon M. K. Birk D. E. (2000). Expression of type XIV collagen in developing chicken tendons: association with assembly and growth of collagen fibrils. Dev. Dyn. 217, 430–439. 10.1002/(SICI)1097-0177(200004)217:4<430::AID-DVDY10>3.0.CO;2-5 10767087

[B140] Yuk J.-S. Lee J. H. Hur J.-Y. Shin J.-H. (2018). The prevalence and treatment pattern of clinically diagnosed pelvic organ prolapse: a Korean national health insurance Database-based cross-sectional study 2009–2015. Sci. Rep. 8, 1334. 10.1038/s41598-018-19692-5 29358718 PMC5778022

[B141] Zhang D. Wu X. Chen J. Lin K. (2017). The development of collagen based composite scaffolds for bone regeneration. Bioact. Mater. 3, 129–138. 10.1016/j.bioactmat.2017.08.004 29744450 PMC5935759

[B142] Zhang Y. Ma Y. Chen J. Wang M. Cao Y. Li L. (2021). Mesenchymal stem cell transplantation for vaginal repair in an ovariectomized rhesus macaque model. Stem Cell Res. Ther. 12, 406. 10.1186/s13287-021-02488-2 34266489 PMC8281669

[B143] Zhou H. Li W. Pan L. Zhu T. Zhou T. Xiao E. (2024). Human extracellular matrix (ECM)-like collagen and its bioactivity. Regen. Biomater. 11, rbae008. 10.1093/rb/rbae008 38545260 PMC10965421

[B144] Zhu B. Li W. Segre C. Lewis R. Janota R. Chi N. (2014). A study of unidirectionally aligned collagen-silk composite fibers and the application in hdpPSC neural differentiation. Microsc. Microanal. 20, 1436–1437. 10.1017/s1431927614008915 25156546

[B145] Zhu B. Li W. Lewis R. V. Segre C. U. Wang R. (2015). E-Spun composite fibers of collagen and dragline silk protein: fiber mechanics, biocompatibility, and application in stem cell differentiation. Biomacromolecules 16, 202–213. 10.1021/bm501403f 25405355 PMC4294589

[B146] Zhu B. Li W. Chi N. Lewis R. V. Osamor J. Wang R. (2017). Optimization of glutaraldehyde vapor treatment for electrospun collagen/silk tissue engineering scaffolds. ACS Omega 2, 2439–2450. 10.1021/acsomega.7b00290 28691110 PMC5494641

